# Supplementary material for: Community’s experience and perceptions of maternal health services across the continuum of care in Ethiopia: A qualitative study
Source: PLoS One. 2021 Aug 4;16(8):e0255404. doi: 10.1371/journal.pone.0255404 (PMC8336848; doi:10.1371/journal.pone.0255404)
Supplement: S1 Appendix — This is an in-depth interview guide we used to interview the participants in our study. (DOCX) [file pone.0255404.s001.docx]

## In-depth Interview Guide (English)

| **I** | **Section I: Identification** | |
| --- | --- | --- |
| 1 | Questionnaire ID | **____________________________** |
| 2 | Area Identification | **____________________________** |
| 3 | Name of Woreda | **____________________________** |
| 4 | Name of Kebele | **____________________________________** |
| 5 | Name of moderator | **_______________________________** |
| 6 | Name of note taker | **_________________________________** |
| 7 | Date of discussion | **_______________________________** |
| 8 | Start time: | **______:________** |
| 9 | End time: | **____:______** |

| **II** | **Section II: Participant Demographic Intake Sheet** | | | | | |
| --- | --- | --- | --- | --- | --- | --- |
| 1 | Participant code |  |  |  |  |  |
| 2 | Age |  |  |  |  |  |
| 3 | Religion |  |  |  |  |  |
| 4 | Marital status |  |  |  |  |  |
| 5 | Are you employed? (Yes/No) |  |  |  |  |  |
| 6 | Educational level |  |  |  |  |  |
| **For recently delivered women** | | | | | | |
| 7 | Gravidity |  |  |  |  |  |
| 8 | Parity |  |  |  |  |  |
| 9 | Place of delivery in last pregnancy (facility/home) |  |  |  |  |  |

**Interview guide**

|  | **For all participants** | | |
| --- | --- | --- | --- |
|  | **Antepartum** | **Intrapartum** | **Postpartum** |
| **The practice of ANC, facility delivery, and PNC services** | | | |
| 1 | How early do women go for ANC? **Probe** why do they go at that time? Why earlier or later? |  | How early do women go for PNC? **Probe** why do they go at that time? Why earlier or later? |
| 2 | How often do they go to ANC? **Probe** why do they go at that time? |  | How often do they go for PNC? **Probe** why do they go at that time? |
| 3 | Do women think skilled attendance during pregnancy helps their pregnancy? | Do women think skilled attendance during childbirth helps themselves and their babies? | Do women think skilled attendance during postpartum helps their babies and themselves? |
| **Reasons for use of ANC, facility delivery and PNC** | | | |
| 4 | Explain factors that would motivate women to utilize ANC service in their pregnancy | Explain factors that would motivate women to utilize delivery service in their pregnancy  **Probe** for reasons for using continuum of care | Explain factors that would motivate women to utilize PNC service in their pregnancy  **Probe** for reasons for using continuum of care |
| **Barriers for attending ANC, facility delivery and PNC use** | | | |
| 5 | If women do not go for ANC, what are their reasons? What are barriers to accessing ANC?  **Probe** for;   1. Financial barriers and opportunity costs 2. Distance and access 3. Socio-cultural 4. Quality of care | If women deliver at home, what are their reasons? Explain the constraints that influenced women to utilize facility delivery services?  **Probe** for;   1. Financial barriers and opportunity costs 2. Distance and access 3. Socio-cultural 4. Quality of care and non-dignified care | If women don't go for post-natal care, what are their reasons? What are barriers to accessing PNC?  **Probe** for;   1. Financial barriers and opportunity costs 2. Distance and access or lack of service 3. Socio-cultural 4. Quality of care |
| **Reasons for discontinuation across the continuum** | | | |
| 6 | Why do women go to the facility for first ANC, but discontinue for subsequent ANC visits?  **Probe** for;   1. Financial barriers and opportunity costs 2. Distance and access 3. Socio-cultural 4. Quality of care | Why do women go to the facility for ANC, yet mostly deliver at home?  **Probe** for;   1. Financial barriers and opportunity costs 2. Distance and access 3. Socio-cultural 4. Quality of care and non-dignified care | Why do women go to the delivery at the facility, yet mostly don’t receive PNC?  Explain the obstacles influenced women to utilize skilled care during pregnancy, childbirth, and postpartum in your community?  **Probe** for;   1. Financial barriers and opportunity costs 2. Distance and access 3. Socio-cultural 4. Quality of care |
| 7 | In your opinion, what should be improved regarding ANC services? | In your opinion, what should be improved regarding facility delivery services? Continuity of care? | In your opinion, what should be improved regarding PNC services? Continuum of care? |
| **Traditional practices during pregnancy, childbirth and postnatal period** | | | |
| 8 | Can you tell us about the traditional practices and beliefs during pregnancy, delivery and postnatal period in your community? | | |
| 9 | Do you think these traditional beliefs, religious practices, and cultural norms affect mothers to use care during pregnancy, delivery, and postpartum period in your community? Explain how and why? | | |
| 10 | How do you see community volunteers/TBAs and health professionals and maternal health services provided to the community? | | |
|  | **For recently delivered mothers only** | | |
| 11 | How do you rate the quality of care you received during ANC follow-up? What kinds of services do you receive in ANC? Are you satisfied? | How do you rate the quality of care you received from the facility during childbirth? What kinds of services do you receive in childbirth? Are they satisfied? | How do you rate the quality of care you received during PNC? What kinds of services do you receive in PNC? Are they satisfied? |
| 12 | If the mother received ANC;  **Ask:** Explain factors that motivate you to utilize ANC service in their pregnancy | If delivered in health facility;  **Ask:** Explain factors that motivate you to deliver in health facility | If use PNC:  **Ask:** Explain factors that motivate you to utilize PNC service |
| 13 | If the mother did not go for ANC;  **Ask**: what are the reasons not attending ANC services? | For home delivered mothers; **Ask:** what do she think are the obstacles when accessing a health care facility? Her reasons for discontinuation? | If women do not go for PNC;  **Ask:**   1. what are the reasons for not getting PNC? 2. what are the reasons for discontinuation? |
| 14 | Explain the support you get from the community to and decision making on health services during pregnancy. delivery and postnatal period | | |
| 15 | Explain us your experiences relating to the utilization of ANC, birth, and PNC care provided by skilled birth attendants. Prove for;   1. their interactions with skilled birth attendants during ANC, delivery, and PNC 2. their confidence in skilled birth attendants’ abilities, and 3. respect and compassion of attendants ( respect for the traditional beliefs of the women, etc) | | |
|  | **For community and religious leaders and community volunteers only** | | |
| **Community perceptions about health providers and maternal health programs** | | | |
| 16 | How the community see the maternal health programs and health professionals? Tell me the perception about maternal health care services. Perception about different care providers. | | |
| 17 | What efforts your community made to increase maternal health service in your community? | | |

**Thank you for your participation!!!**
