# Supplementary material for: Community’s experience and perceptions of maternal health services across the continuum of care in Ethiopia: A qualitative study
Source: PLoS One. 2021 Aug 4;16(8):e0255404. doi: 10.1371/journal.pone.0255404 (PMC8336848; doi:10.1371/journal.pone.0255404)
Supplement: S2 Appendix — This is a focus group discussion guide we used to elicit discussion with respondents. (DOCX) [file pone.0255404.s002.docx]

## Focus Group Discussion Guide (English)

| **I** | **Section I: Identification** | |
| --- | --- | --- |
| 1 | Questionnaire ID | **____________________________** |
| 2 | Area Identification | **____________________________** |
| 3 | Name of Woreda | **____________________________** |
| 4 | Name of Kebele | **____________________________________** |
| 5 | Name of moderator | **_______________________________** |
| 6 | Name of note taker | **_________________________________** |
| 7 | Date of discussion | **_______________________________** |
| 8 | Start time: | **______:________** |
| 9 | End time: | **____:______** |

**Discussion guide**

|  | **For all participants** | |  |
| --- | --- | --- | --- |
| **Practice of ANC, facility delivery, and PNC services** | | |  |
| 1 | How early do women go for ANC? How early do women go for PNC? Why do they go at that time? Why earlier or later? | |  |
| 2 | What kinds of services do they receive in ANC, childbirth and PNC? Are they satisfied? | |  |
| 3 | Do women think skilled attendance during pregnancy, childbirth, and PNC helps their pregnancy, babies and themselves? | |  |
| **Reasons for use of ANC, facility delivery and PNC** | | |  |
| 4 | Explain factors that would motivate women to utilize ANC service, institutional delivery, and PNC  **Probe** for reasons for using continuum of care | |  |
| **Barriers for attending ANC, facility delivery and PNC use** | | |  |
| 5 | If women do not go for ANC, delivery, and PNC what are their reasons?  Explain the obstacles influenced women to utilize ANC, PNC, facility delivery services in your community? Obstacles using different care providers?  **Probe** for financial barriers and opportunity costs, geographic barriers, socio-cultural barriers and quality of care barriers | |  |
| **Reasons for discontinuation** | | |  |
| 6 | 1. Why do women go to the facility for first ANC, but discontinue for subsequent ANC visits? Are all mothers abiding to their appointment dates for ANC consultation? 2. Why do women go to the facility for ANC, yet mostly deliver at home? 3. Why do women go to the delivery at the facility, yet mostly don’t receive PNC? Are all mothers abiding to their appointment dates for PNC consultation?   **Probe** for financial barriers and opportunity costs, geographic barriers, socio-cultural barriers and quality of care barriers | |  |
| 7 | What costs do you think involved for pregnant women to attend ANC, facility delivery and PNC services? | |  |
| 8 | In your opinion, what should be improved regarding **ANC, facility delivery, and PNC** | |  |
| **Traditional practices during pregnancy, childbirth and postnatal period** | | |  |
| 9 | Can you tell us about the traditional practices and beliefs during **pregnancy, delivery and postnatal period** in your community? | |  |
| 10 | Do you think these traditional beliefs, religious practices, and cultural norms affect mothers to use care during pregnancy, delivery, and postpartum period in your community? Explain how and why? | | |
| 11 | How do you see community volunteers/TBAs, health professionals and maternal health services provided to the community? | |  |
|  | **For recently delivered mothers only** | |  |
| 12 | Explain factors that motivate you to utilize ANC service, institutional delivery, and PNC  **Probe** for reasons for using continuum of care | |  |
| 13 | 1. In your opinion what are reasons for not attending ANC services? 2. For home delivered mothers, what do you think are the obstacles when accessing a health care facility? **Probe** for discontinuation after receiving ANC 3. If women do not go for PNC, what are their reasons? **Probe** for discontinuation after receiving ANC and/or facility delivery | |  |
| 14 | Explain us your experiences relating to the utilization of ANC, birth, and PNC care provided by skilled birth attendants. Prove for;   - their interactions with skilled birth attendants during ANC, delivery, and PNC - their confidence in skilled birth attendants’ abilities, and - respect and compassion of attendants ( respect for the traditional beliefs of the women, etc) | |  |
| 15 | Explain the support you get from the community to and decision making on health services during **pregnancy. delivery and postnatal period** | |  |
|  | **For community and religious leaders and community volunteers only** | |  |
| **Community perceptions about health providers and maternal health programs** | |  |  |
| 16 | How the community see the maternal health programs and health professionals? Tell me the perception about maternal health care services. Perception about different care providers. |  |  |
| 17 | What efforts your community made to increase maternal health service in your community? |  |  |

**Thank you for your participation!!!**
