# Supplementary material for: Community’s experience and perceptions of maternal health services across the continuum of care in Ethiopia: A qualitative study
Source: PLoS One. 2021 Aug 4;16(8):e0255404. doi: 10.1371/journal.pone.0255404 (PMC8336848; doi:10.1371/journal.pone.0255404)
Supplement: S3 Appendix — This file contains in-depth interview transcripts used for this analysis. (DOC) [file pone.0255404.s003.doc]

# IDI Transcripts

# IDI with a community leader_Jabi_191105_0816

| **I** | **Section I: Identification** | |
| --- | --- | --- |
| 1 | Questionnaire ID | **191105_0816_ayzoh** |
| 2 | Area Identification | **West Gojjam Zone** |
| 3 | Name of Woreda | **Jabi Tehinan** |
| 4 | Name of Kebele | **Jiga Zuria** |
| 5 | Name of moderator | **Chalachew** |
| 6 | Name of note taker | **Ayizohibel Adamu** |
| 7 | Date of discussion |  |
| 8 | Start time: | **______:________** |
| 9 | End time: | **18:32** |

| **II** | **Section II: Participant Demographic Intake Sheet** | | | | | |
| --- | --- | --- | --- | --- | --- | --- |
| 1 | Participant code |  |  |  |  |  |
| 2 | Age | 60 |  |  |  |  |
| 3 | Religion | Orthodox Christian |  |  |  |  |
| 4 | Marital status | Married |  |  |  |  |
| 5 | Are you employed? (Yes/No) | No |  |  |  |  |
| 6 | Educational level | Not educated |  |  |  |  |

## Antepartum

***The practice of ANC, facility delivery, and PNC services***

1. How early do women go for ANC? **Probe** why do they go at that time? Why earlier or later?

Respondent: Usually the start ANC services after one month of missing their last menstrual period. This is actually what I observed even if we stopped giving birth because of age.

1. How often do they go to ANC? **Probe** why do they go at that time?

Respondent: Then pregnant mothers will have monthly follow up at the health center or hospital

1. Do women think skilled attendance during pregnancy helps their pregnancy?

Respondent: Yes. In the earlier time before health service delivery has been expanded as such, mother have had delivered their babies at home which in turn cause severe bleeding (SERAKIAN). We had bad practices like gun shout, immersing the bleeding mother in water and the likes. But now the mothers have proper follow up and appointments for institutional delivery. If labour begins at home and if she bleeds immediately send to the health centre or hospital

Health care workers provide health education and the community awareness/knowledge has been improved as the result there are no people as such who opposed the services like ANC, Delivery and PNC services

***Reasons for use of ANC, facility delivery and PNC***

1. Explain factors that would motivate women to utilize ANC service in their pregnancy

Respondent: To get better health, since they observed what happening from lack of ANC follow up and home delivery

***Barriers for attending ANC, facility delivery and PNC use***

1. If women do not go for ANC, what are their reasons? What are barriers to accessing ANC? **Probe** for; Financial barriers and opportunity costs, Distance and access, Socio-cultural, Quality of care

Respondent: I don’t know, No problems

***Reasons for discontinuation across the continuum***

1. Why do women go to the facility for first ANC, but discontinue for subsequent ANC visits? **Probe** for; Financial barriers and opportunity costs, Distance and access, socio-cultural, Quality of care

Respondent: I don’t know mothers who interrupted ANC follow up.

1. In your opinion, what should be improved regarding ANC services?

Respondent: access shall be at health post, there are some far kebeles which have difficulty of getting these services on time

So bed services, medicine/ drug supply and service in general. Because the population in this town is increasing but the service potential is less.

Health centre is needed for this kebele (high town population)

***Traditional practices during pregnancy, childbirth and postnatal period***

1. Can you tell us about the traditional practices and beliefs during pregnancy, delivery and postnatal period in your community?

Respondent: Bye now it is advised about good nutrition according to the resources they have. But I told you before there are uvulectomy traditional practices

As I told you earlier there were some malpractices had been conducted while delivery if the mother bleeds severely, we call it Serakian. The malpractices were gun shout, immersing the bleeding mother in water bodies and the likes. But now these practices and other malpractice which I did not tell you before that is female genitalia cutting are ceased. However, there are still some practices like uvulectomy is practicing in few villages and people. There is one man at the kebele town of Jiga, people go there for uvulectomy when their children get sick.

1. Do you think these traditional beliefs, religious practices, and cultural norms affect mothers to use care during pregnancy, delivery, and postpartum period in your community? Explain how and why?

Respondent: I don’t think it is good. Previously it was believed that unless girls have got circumcised (Female Genitalia cutting) they would be non-ethical girls. But this thinking is now stopped

These practices happing were years back when people have low awareness about the problem. Now we get trained from malpractices risks and usually the health workers teach about good health practices and utilization of service delivery at health facility.

As I told you, I understand the traditional are harmful and I am advising my children and neighbours to attend ANC, delivery and PNC at health centre or hospital. That is why traditional practices during pregnancy, delivery and PNC ceased because of community awareness

1. How do you see community volunteers/TBAs and health professionals and maternal health services provided to the community?

Respondent: The community has good perception about service provision

## Intrapartum

***The practice of facility delivery***

1. Do women think skilled attendance during childbirth helps themselves and their babies?

***Reasons for use of facility delivery***

1. Explain factors that would motivate women to utilize delivery service in their pregnancy **Probe** for reasons for using continuum of care

Respondent: To get better health, since they observed what happening from lack of ANC follow up and home delivery

***Barriers for attending ANC, facility delivery and PNC use***

1. If women deliver at home, what are their reasons? Explain the constraints that influenced women to utilize facility delivery services? **Probe** for; Financial barriers and opportunity costs, Distance and access, Socio-cultural, Quality of care and non-dignified care

Respondent: Some women delivery at home but majority give birth with skilled delivery

But few mothers give birth at home due to emergency, few others also give birth due to cultural believes, “Mariam helps you in while you deliver and brings birth. Esua yekuaterchiwn esua tifetawalech.”

***Reasons for discontinuation across the continuum***

1. Why do women go to the facility for ANC, yet mostly deliver at home? Probe for; Financial barriers and opportunity costs, Distance and access, Socio-cultural, Quality of care and non-dignified care
2. In your opinion, what should be improved regarding facility delivery services? Continuity of care?

## Postpartum

***The practice of ANC, facility delivery, and PNC services***

1. How early do women go for PNC? **Probe** why do they go at that time? Why earlier or later?

Respondent: Mothers have postnatal follow up after delivery in the hospital. The community has annual contribution of different crop seeds which will be used for serving mothers at postnatal services admitted in a hospital and or health centre.

# IDI with a recently delivered woman_Dembecha_191103_0758

| **I** | **Section I: Identification** | |
| --- | --- | --- |
| 1 | Questionnaire ID | **191103_0758** |
| 2 | Area Identification |  |
| 3 | Name of Woreda | **____________________________** |
| 4 | Name of Kebele | **____________________________________** |
| 5 | Name of moderator | **CB** |
| 6 | Name of note taker | **AA** |
| 7 | Date of discussion | **_______________________________** |
| 8 | Start time: | **Afternoon** |
| 9 | End time: | **24: 24** |

| **II** | **Section II: Participant Demographic Intake Sheet** | | | | | |
| --- | --- | --- | --- | --- | --- | --- |
| 1 | Participant code |  |  |  |  |  |
| 2 | Age |  |  |  |  |  |
| 3 | Religion |  |  |  |  |  |
| 4 | Marital status |  |  |  |  |  |
| 5 | Are you employed? (Yes/No) |  |  |  |  |  |
| 6 | Educational level |  |  |  |  |  |

## Antepartum

***The practice of ANC, facility delivery, and PNC services***

1. How early do women go for ANC? **Probe** why do they go at that time? Why earlier or later?

Respondent: mothers started ANC about 3 months and continue receiving the service monthly, we started at 3 months.

Interviewer: Why don’t they started early? What do u think are the reasons? Why u and ur neighbour don’t start before 3 months?

Respondent: First we [me and my neibour] tested our pregnancy from private [clinic] and started ANC follow up at the HC. Then we went monthly, then latter biweekly till our delivery.

Do u know mothers who started ANC early?

Respondent: I don’t think there are women who started before 3 months.

Interviewer: Why do think are the reasons:

Respondent: May be, here most of us are farmers; not educated.

Respondent: probably, they may not know their LMP; they started booking after they received pregnancy test at the HC. That is why they elapsed one or two months to start ANC booking. After 3 months, they continued their follow-up till delivery.

Interviewer: are there mothers who started late?

Respondent: ya, particularly those who lived far.

Interviewer: Wht do think are the reasons?

Respondent: I don’t know. But the health workers inquire us why u are not following your pregnancy?

1. How often do they go to ANC? **Probe** why do they go at that time?

Respondent: we went on monthly basis. They appointed us to follow monthly and then biweekly when we get term

1. Do women think skilled attendance during pregnancy helps their pregnancy?

Respondent:

***Reasons for use of ANC, facility delivery and PNC***

1. Explain factors that would motivate women to utilize ANC service in their pregnancy

***Barriers for attending ANC, facility delivery and PNC use***

1. If women do not go for ANC, what are their reasons? What are barriers to accessing ANC? **Probe** for; Financial barriers and opportunity costs, Distance and access, Socio-cultural, Quality of care

I don’t know. But I think people around here concerns more on their job than their health. I think it is this.

***Reasons for discontinuation across the continuum***

1. Why do women go to the facility for first ANC, but discontinue for subsequent ANC visits? **Probe** for; Financial barriers and opportunity costs, Distance and access, socio-cultural, Quality of care

There are women who interrupts their monthly follow-up. For instance, I interrupted for one month because I went to other villages. And they told me I could also follow there.

1. In your opinion, what should be improved regarding ANC services?

No problem, except the distance. We don’t get transport.

***Traditional practices during pregnancy, childbirth and postnatal period***

1. Can you tell us about the traditional practices and beliefs during pregnancy, delivery and postnatal period in your community?
2. Do you think these traditional beliefs, religious practices, and cultural norms affect mothers to use care during pregnancy, delivery, and postpartum period in your community? Explain how and why?
3. How do you see community volunteers/TBAs and health professionals and maternal health services provided to the community?

## Intrapartum

***The practice of facility delivery***

1. Do women think skilled attendance during childbirth helps themselves and their babies?

Interviewer: How do mothers see give birth at health facility?

Respondent: ya, it is important. They helped us; support us during labour. It helps us no problem encountered us as a result of the pregnancy

***Reasons for use of facility delivery***

1. Explain factors that would motivate women to utilize delivery service in their pregnancy **Probe** for reasons for using continuum of care

Respondent: It is said, if you give birth at home, you will be charged 500-600 birr. They counsel us; their counseling is good. They also refer us if it is beyond their scope. We believed; it is good for us our baby

***Barriers for attending ANC, facility delivery and PNC use***

1. If women deliver at home, what are their reasons? Explain the constraints that influenced women to utilize facility delivery services? **Probe** for; Financial barriers and opportunity costs, Distance and access, Socio-cultural, Quality of care and non-dignified care

Respondent: I don’t think there are women who give birth at home. All are delivering at HC. All women when labor started, we called to ambualce and went to Hc and deliver there.

Interviewer: Would there be due to distance or cost factor, women may deliver at hme?

Respondent: I don’t think so. May be those in remote areas, but there is no women delivering home around this village.

***Reasons for discontinuation across the continuum***

1. Why do women go to the facility for ANC, yet mostly deliver at home? Probe for; Financial barriers and opportunity costs, Distance and access, Socio-cultural, Quality of care and non-dignified care

There is no women delivering at home. Even those in remote villages are also delivering at HC.

1. In your opinion, what should be improved regarding facility delivery services? Continuity of care?

Nothing. It is good. They support us during labor

***Traditional practices during pregnancy, childbirth and postnatal period***

1. Can you tell us about the traditional practices and beliefs during pregnancy, delivery and postnatal period in your community?
2. Do you think these traditional beliefs, religious practices, and cultural norms affect mothers to use care during pregnancy, delivery, and postpartum period in your community? Explain how and why?
3. How do you see community volunteers/TBAs and health professionals and maternal health services provided to the community?
4. Explain us your experiences relating to the utilization of ANC, birth, and PNC care provided by skilled birth attendants. Prove for;

• their interactions with skilled birth attendants during ANC, delivery, and PNC

• their confidence in skilled birth attendants’ abilities, and

• respect and compassion of attendants ( respect for the traditional beliefs of the women, etc)

Respondent: they helped us. They followed us. They are good. They provide us the necessary support

## Postpartum

***The practice of ANC, facility delivery, and PNC services***

1. How early do women go for PNC? **Probe** why do they go at that time? Why earlier or later?

Interviewer: Do u go to facility for PNC?

Respondent: No, it is for the baby girl, not for me.

Don’t u go to facility for your health?

Respondent: No, I didn’t go/

Interviewer: Don’t they told u to back for PNC?

Respondent: No, they don’t tell me to go back. They instructed me how to care for the perineum and to get back for birth control in 45 days, otherwise, they don’t tell me to back for PNC

Interviewer: how may hours did u saty after delivery:

Respondent: I sated for 8 hours

Interviewer: Was there any health worker who come to home and follow u?

Respondent: No, no one

1. How often do they go for PNC? **Probe** why do they go at that time?
2. Do women think skilled attendance during postpartum helps their babies and themselves?

Respondent: if there is a follow-up for our kids, it would be good. For us, no need; not necessary.

Interviewer: explain me Why? Or what are u saying when u say not necessary?

Respondent: I don’t think it is important. [laughing] I don’t get sick after delivery.

***Reasons for use of ANC, facility delivery and PNC***

1. Explain factors that would motivate women to utilize PNC service in their pregnancy

***Barriers for attending ANC, facility delivery and PNC use***

1. If women don't go for post-natal care, what are their reasons? What are barriers to accessing PNC? **Probe** for; Financial barriers and opportunity costs, Distance and access or lack of service, Socio-cultural, Quality of care

***Reasons for discontinuation across the continuum***

1. Why do women go to the delivery at the facility, yet mostly don’t receive PNC? Explain the obstacles influenced women to utilize skilled care during pregnancy, childbirth, and postpartum in your community? **Probe** for; financial barriers and opportunity costs, Distance and access, Socio-cultural, Quality of care.
2. In your opinion, what should be improved regarding PNC services? Continuum of care?

***Traditional practices during pregnancy, childbirth and postnatal period***

1. Can you tell us about the traditional practices and beliefs during pregnancy, delivery and postnatal period in your community?
2. Do you think these traditional beliefs, religious practices, and cultural norms affect mothers to use care during pregnancy, delivery, and postpartum period in your community? Explain how and why?

Respondent: uvulectomy. We believed it would kill our newborns. We paid 150 birr. This is our problem. I think it is helpful. We fear they may die. Providers told us not to do so.

1. How do you see community volunteers/TBAs and health professionals and maternal health services provided to the community?

They are nice; they helped us.

# IDI with Community leader_Jabi_191102_0938

| **I** | **Section I: Identification** | |
| --- | --- | --- |
| 1 | Questionnaire ID | **191102_0938** |
| 2 | Area Identification | **Jabi** |
| 3 | Name of Woreda | **____________________________** |
| 4 | Name of Kebele | **____________________________________** |
| 5 | Name of moderator | **CB** |
| 6 | Name of note taker | **AA** |
| 7 | Date of discussion | **_______________________________** |
| 8 | Start time: | **______:________** |
| 9 | End time: | **34:50** |

| **II** | **Section II: Participant Demographic Intake Sheet** | | | | | |
| --- | --- | --- | --- | --- | --- | --- |
| 1 | Participant code |  |  |  |  |  |
| 2 | Age |  |  |  |  |  |
| 3 | Religion |  |  |  |  |  |
| 4 | Marital status |  |  |  |  |  |
| 5 | Are you employed? (Yes/No) |  |  |  |  |  |
| 6 | Educational level |  |  |  |  |  |

## Antepartum

***The practice of ANC, facility delivery, and PNC services***

1. How early do women go for ANC? **Probe** why do they go at that time? Why earlier or later?

Respondent: mothers started ANC at 3 months and continue receiving the service at the HC till their delivery. And some deliver at HC; some deliver at home.

Interviewer: Why don’t they started early?

Respondent: probably, they may not know their LMP; they started booking after they received pregnancy test at the HC. That is why they elapsed one or two months to start ANC booking. After 3 months, they continued their follow-up till delivery.

Interviewer: are there mothers who started late?

Respondent: nowadays, HDAs are mobilizing/reminding mothers to go to HC and start ANC. As such, mothers started receiving vaccinations. Otherwise, mothers usually be reluctant to start. Ya, they went monthly. Nowadays, they are going to attend ANC.

1. How often do they go to ANC? **Probe** why do they go at that time?
2. Do women think skilled attendance during pregnancy helps their pregnancy?

Respondent: Ya, they appreciated very much. In earlier times, it was without knowledge. There are mothers who stayed there for a month. They received care, food, etc. they acknowledged very much for this.

***Reasons for use of ANC, facility delivery and PNC***

1. Explain factors that would motivate women to utilize ANC service in their pregnancy

Respondent: in-depth counseling by providers going at gote level (guben). Those who adopts the counseling attends ANC, delivery at facility. But there are some women who still deliver at home.

***Barriers for attending ANC, facility delivery and PNC use***

1. If women do not go for ANC, what are their reasons? What are barriers to accessing ANC? **Probe** for; Financial barriers and opportunity costs, Distance and access, Socio-cultural, Quality of care

Respondent: Still there are some mothers who wants to give birth at home because of the old tradition. But HDAs and health workers are motivating them to attend ANC and give birth at HF. There are also wise women who followed their pregnancy, stayed at facility (MWH) before labor, and delivered at facility. These women are telling their stories (importance of attending and delivering at facility) to others.

While I was a team leader and discuss with them, they said, I don’t like to disclose my secrets. Cost is not an issue. We used CBHI. No issue regarding acceptance of the service.

***Reasons for discontinuation across the continuum***

1. Why do women go to the facility for first ANC, but discontinue for subsequent ANC visits? **Probe** for; Financial barriers and opportunity costs, Distance and access, socio-cultural, Quality of care

**Already addressed in question 5 above.**

1. In your opinion, what should be improved regarding ANC services?

***Traditional practices during pregnancy, childbirth and postnatal period***

1. Can you tell us about the traditional practices and beliefs during pregnancy, delivery and postnatal period in your community?
2. Do you think these traditional beliefs, religious practices, and cultural norms affect mothers to use care during pregnancy, delivery, and postpartum period in your community? Explain how and why?
3. How do you see community volunteers/TBAs and health professionals and maternal health services provided to the community?

## Intrapartum

***The practice of facility delivery***

1. Do women think skilled attendance during childbirth helps themselves and their babies?

Interviewer: How do mothers see give birth at health facility?

Respondent: earlier times, it was 2-3 days labouring at home without support. Nowadays, they feel it is for them to

***Reasons for use of facility delivery***

1. Explain factors that would motivate women to utilize delivery service in their pregnancy **Probe** for reasons for using continuum of care

***Barriers for attending ANC, facility delivery and PNC use***

1. If women deliver at home, what are their reasons? Explain the constraints that influenced women to utilize facility delivery services? **Probe** for; Financial barriers and opportunity costs, Distance and access, Socio-cultural, Quality of care and non-dignified care

Respondent: Those who deliver at home, said they want to deliver at home as per the tradition. because they don’t want to expose their privacy. They want to follow the tradition. There are women who deliver at home without disclosing their pregnancy. It is about the privacy. It is not distance or cost is an issue.

***Reasons for discontinuation across the continuum***

1. Why do women go to the facility for ANC, yet mostly deliver at home? Probe for; Financial barriers and opportunity costs, Distance and access, Socio-cultural, Quality of care and non-dignified care

Respondent: Women are not discontinuing once they stated. WDAs are motivating them to continue their follow-up.

Interviewer: are there mothers who attend ANC but gave birth at home?

Respondent: some may give birth at home. There are mothers who have easy labor and give birth at home unintentionally (despite their plan to deliver at facility). It is about the easy of labor. But once they started ANC they would continue to deliver at facility.

1. In your opinion, what should be improved regarding facility delivery services? Continuity of care?

Respondent: There are mothers who complain about the facility. They said after delivery the discharge and left us unattended. They asked why the ambulance return us home once he took us to the facility! Those who have money they back using contract transport. But those who don’t have money, they travelled long distances carrying newborn. There is a problem regarding ambulance use.

***Traditional practices during pregnancy, childbirth and postnatal period***

1. Can you tell us about the traditional practices and beliefs during pregnancy, delivery and postnatal period in your community?

Respondent: they said I don’t want to expose myself going there. they preferred to have attended by tTBAs. They afraid of exposing their reproductive organ. They preferred to have training to TBAs and attended by them at home or facility. Because she has such experiences (Ye setoch limid yalat) and is not exposing them. They afraid male attendants.

1. Do you think these traditional beliefs, religious practices, and cultural norms affect mothers to use care during pregnancy, delivery, and postpartum period in your community? Explain how and why?

Respondent: This hinders women not to attend facility delivery. They said, endew setoch behonu enkua behone, sewnetachin yishemakekal yilalu. When we told them why u care what is needed is saving your life, they replied us we afraid, sewnetachin yishemakekal yilalu.

1. How do you see community volunteers/TBAs and health professionals and maternal health services provided to the community?

Respondent: their services, people acknowledged. They follow well. They provide services well. We don’t encounter problems on our mothers and children since this HC is established.

Interviewer: what is communities’ contribution

Respondent: there is priority to work. It is the WDAs who motivate mothers and took them to facility. But fathers are not carrying about attending pregnancy or delivering at facility they don’t understand mother’s problem in-depth; They prioritized their work. They prefer to engage mothers to work

## Postpartum

***The practice of ANC, facility delivery, and PNC services***

1. How early do women go for PNC? **Probe** why do they go at that time? Why earlier or later?

Interviewer: Do women go to facility for PNC?

Respondent: Ya, they are following. Mothers feel, probably, if there is any illness from my side not to transmit to the newborn, they went and follow their health.

Interviewer: mothers counselled for ANC and delivery. Do they also counsel for PNC? They are counselled to attend [vaccination] monthly after delivery. Mothers are attending vaccination on monthly basis.

1. How often do they go for PNC? **Probe** why do they go at that time?
2. Do women think skilled attendance during postpartum helps their babies and themselves?

***Reasons for use of ANC, facility delivery and PNC***

1. Explain factors that would motivate women to utilize PNC service in their pregnancy

***Barriers for attending ANC, facility delivery and PNC use***

1. If women don't go for post-natal care, what are their reasons? What are barriers to accessing PNC? **Probe** for; Financial barriers and opportunity costs, Distance and access or lack of service, Socio-cultural, Quality of care

***Reasons for discontinuation across the continuum***

1. Why do women go to the delivery at the facility, yet mostly don’t receive PNC? Explain the obstacles influenced women to utilize skilled care during pregnancy, childbirth, and postpartum in your community? **Probe** for; financial barriers and opportunity costs, Distance and access, Socio-cultural, Quality of care.
2. In your opinion, what should be improved regarding PNC services? Continuum of care?

***Traditional practices during pregnancy, childbirth and postnatal period***

1. Can you tell us about the traditional practices and beliefs during pregnancy, delivery and postnatal period in your community?
2. Do you think these traditional beliefs, religious practices, and cultural norms affect mothers to use care during pregnancy, delivery, and postpartum period in your community? Explain how and why?

Respondent: No, there are no tradition or belief related factors hindering mothers to access the PNC services.

1. How do you see community volunteers/TBAs and health professionals and maternal health services provided to the community?

# 4. IDI with HDA_Tebelima_Womberima_191102_1114

| **I** | **Section I: Identification** | |
| --- | --- | --- |
| 1 | Questionnaire ID | **191102_1114** |
| 2 | Area Identification | **Agumam Tebelima** |
| 3 | Name of Woreda | **____________________________** |
| 4 | Name of Kebele | **____________________________________** |
| 5 | Name of moderator | **CB** |
| 6 | Name of note taker | **AA** |
| 7 | Date of discussion | **_______________________________** |
| 8 | Start time: | **______:________** |
| 9 | End time: | **____:______** |

| **II** | **Section II: Participant Demographic Intake Sheet** | | | | | |
| --- | --- | --- | --- | --- | --- | --- |
| 1 | Participant code | 01 |  |  |  |  |
| 2 | Age |  |  |  |  |  |
| 3 | Religion |  |  |  |  |  |
| 4 | Marital status |  |  |  |  |  |
| 5 | Are you employed? (Yes/No) |  |  |  |  |  |
| 6 | Educational level |  |  |  |  |  |

## Antepartum

***The practice of ANC, facility delivery, and PNC services***

1. How early do women go for ANC? **Probe** why do they go at that time? Why earlier or later?

Interviewer: tell us about your role as HDA in the community?

Respondent: I am residing in Agumame Tebelima, and I am a WDA and have been working on this role ever since. The WDAs in the different ‘gotes’ are many in number and for instance in our ‘gote’; we are four in number and we have been working on mothers. Previously, there issue did not have much attention, however, currently we are much focused and at more depth. The WDAs now knows exactly what a pregnant should do before and after her pregnancy. We also meet and discuss with pregnant mothers every month and the 1-5 network also meets every fortnight on Sunday. We discuss on the frequency of ANC visit for a pregnant women free of any sickness advised to do at least four times

1. How often do they go to ANC? **Probe** why do they go at that time?

Interviewer: How often do pregnant mothers start their ANC in these locality?

Respondent: most of the time it is after their three months if they don’t feel any pain.

Interviewer: Why do you think it is the reason for this?

Respondent: may be because they don’t feel anything before this time and also because they know for sure they are pregnant at this time and then after their three month they go to the HFs for ANC. I think they don’t believe they could be pregnant before three months.

Interviewer: for how long and how frequent do they follow their ANC?

Respondent: If she feels sick, she might visit the HC before her schedule for ANC and might visit four or five times. However, if she is not sick, she might make the visit four times.

1. Do women think skilled attendance during pregnancy helps their pregnancy?

Respondent: Yes, it is helpful for a mother to make ANC until her delivery because she might develop Anemia or hypertension during the course of her pregnancy and also there is a test for HIV; and if she is not checked for this and not consulted about this at a HC, she might suffer from complications if she, for instance develops anemia during her labor.

Interviewer: What do you think benefits the pregnant mother when she delivers through a skilled professional?

Respondent: it will prevent her from facing of any health risks, she might even die otherwise. However, if she delivers at a HF, there is a lot that is made sure for her, and she will not suffer from any bleeding during her labor, and there is also care provided to the newborn, and medications as well.

***Reasons for use of ANC, facility delivery and PNC***

1. Explain factors that would motivate women to utilize ANC service in their pregnancy

***Barriers for attending ANC, facility delivery and PNC use***

1. If women do not go for ANC, what are their reasons? What are barriers to accessing ANC? **Probe** for; Financial barriers and opportunity costs, Distance and access, Socio-cultural, Quality of care

Interviewer: Are there any pregnant mothers who don’t make ANC visits?

Respondent: No there are not, there is no a single pregnant mother who are not making ANC under my catchment. This was a happening in previous times, currently there are no pregnant mothers who are not making ANC for sure. In my case for instance, there are 28 pregnant mothers and all are following ANC, even by staying away from their homes for one or two weeks in the HCs waiting room facilities during delivery.

Interviewer: If there are no pregnant mothers who are not making ANC in your catchment, what do you think are their reasons?

Respondent: this is because, there is also an obligatory regulation set on home delivery. There is a punishment that our leadership pays in cash about 500 ETB if a pregnant mother is found delivered at home under its catchment.

Interviewer: what are the reasons for some of the mothers not to make ANC at HFs?

Respondent: It is awareness related gap among some uneducated mothers stuck with previous beliefs and suggesting the change it would make if they deliver at home or don’t make ANC visits. However, the situation has now changed due to the provision of health education and other efforts, and currently, there are mothers who are not making ANC follow ups. This is because the mothers care about the well fare of their babies as well.

***Reasons for discontinuation across the continuum***

1. Why do women go to the facility for first ANC, but discontinue for subsequent ANC visits? **Probe** for; Financial barriers and opportunity costs, Distance and access, socio-cultural, Quality of care

**Already addressed in question 5 above.**

1. In your opinion, what should be improved regarding ANC services?

Respondent: it is better if it continues as in the trend we have come so far just like the ways we have been educated; the monthly discussion, and health education is nice if it resumes the same way. We discuss every month on issues related with ANC and PNC, and the frequencies of the ANC visits when a mother gets pregnant, and all of us women meet regularly. There is a schedule that we meet for our saving purpose and there is not issue that we don’t raise about ANC follow ups; there are women organized under village saving groups which there are a lot of education and the regular mothers conference held every 7^th^ of the month which there are WDAs leading the activity up front.

Interviewer: if there are any challenges raised on the ANC service in the community and any solutions you propose on the service?

Respondent: there are no such kind of gaps or anything they raised on the ANC service. It would just be a onetime experience from newly pregnant mothers when they make their first ANC visits feeling fearful, other than that there are no problems.

***Traditional practices during pregnancy, childbirth and postnatal period***

1. Can you tell us about the traditional practices and beliefs during pregnancy, delivery and postnatal period in your community?

Respondent: there is no any practices or beliefs.

Interviewer: What I mean is for instance, there is a belief in my society, making of a gunfire to keep Satan away of the postpartum mother, an if there are similar practices for the postpartum mothers and related with the delivery?

Respondent: If you are asking me about the things done in the house related with the birth. Well, family members of the postpartum decorates the house, well dressed themselves and warmly receives the postpartum mother when she returns to her house. They also prepare food, organize fest and the neighbors come and share her joy.

Interviewer: what else, for instance, what I heard in this community, sometimes they prefer the newborn not to be seen a stranger, and they also prefer if they are assisted by relatives during delivering than strangers, do you know such beliefs in your community which might discourage from making follow ups to HFs?

Respondent: Be it the relatives or neighbors, once the status of a woman in the community is known to be pregnant, there is a support made to the pregnant mother to ease her from any exhaustion from work overload and help her have a rest until she gives birth.

1. Do you think these traditional beliefs, religious practices, and cultural norms affect mothers to use care during pregnancy, delivery, and postpartum period in your community? Explain how and why?

Respondent: No, there are no tradition or belief related factors hindering mothers to access the ANC services.

1. How do you see community volunteers/TBAs and health professionals and maternal health services provided to the community?

Respondent: The service provided to pregnant mothers by these groups is in the form of porridge and other different supports including from WDAs as well. The pregnant mothers are supported with cereals like Barley from contributions of the HDAs to prepare porridge for the mothers. The practice by WDAs is still there even if now hired professionals are making and provisions porridge to the postpartum mothers in the waiting rooms.

Interviewer: Who contributes the cereals?

Respondent: there are the twenty pregnant mothers under me and those of us who are volunteer contributes one dish of Teff in the name of the pregnant mothers for nourishing after their delivery.

Interviewer: How do you contribute the income used for the waiting room service?

Respondent: The community has discussed and dedicated a land for serving the waiting room with income and now the contribution of cereals is no more a practice and income is generated from the dedicated land in the form of rent. The income collected is used to purchase food items for the waiting room purpose. A ‘GEZIN’ of land is dedicated from each ‘gote’ for this purpose.

Interviewer: How about the service provision from the health care providers?

Respondent: the health care providers are hardworking and providing proper care for the pregnant mothers or the newborns.

Interviewer: How do you the community see the service provided in the community from the health care providers?

Respondent: they understand well and of their efforts. The health provision has been understood very well by the community and the health education provided by the health care providers is well received in the community.

Interviewer: the other thing is in the Ambulance transport service, would there be anything the community suggests for any improvements?

Respondent: No there is no problem.

Interviewer: Is Ambulance available any time for the community wants, and challenges for returning of pregnant mothers after referring to HFs?

Respondent: No there is no problem, they are available whenever they are called and no issues raised with regard to returning mothers to their villages after delivery.

Interviewer: Are there any pushes and encouragement for the community to make timely ANC, delivery or PNC, including you as HDA?

Respondent: the community is provided with health education regarding maternal care by the health care providers and it is well received by the community, and the community respond for anything they are required of committing.

Interviewer: If there is anything you want to additionally mention at the end of our interview, that was not mentioned during the interview?

Respondent: No, I think we raised all the issues.

Interviewer: thank you very for your time, you have given us rich, an elaborated and clear information.

Respondent: my pleasure.

## Intrapartum

***The practice of facility delivery***

1. Do women think skilled attendance during childbirth helps themselves and their babies?

Interviewer: What do you think the advantage delivering by a skilled provider?

Respondent: well, she will benefit from minimizing health related risks. Mothers may even die of bleeding during labor, however, if she is delivered by a skilled provider, she will get a lot of support and proper medication if she gets sick. The mother might not get these advantages available at HFs if she delivers at home, including missing of proper care for the child in case of an unanticipated health outcomes.

***Reasons for use of facility delivery***

1. Explain factors that would motivate women to utilize delivery service in their pregnancy **Probe** for reasons for using continuum of care

Respondent: It is the complete rest they get when they come for delivery at HC from the mothers waiting room service. It is advised for pregnant mothers to stay for one month in the mothers waiting room happy and without engaging in any hard works. The stay at the waiting rooms makes the pregnant mothers happy and free of any workloads, which motivated them to deliver at the health facilities.

***Barriers for attending ANC, facility delivery and PNC use***

1. If women deliver at home, what are their reasons? Explain the constraints that influenced women to utilize facility delivery services? **Probe** for; Financial barriers and opportunity costs, Distance and access, Socio-cultural, Quality of care and non-dignified care

Respondent: it was in the previous times that mothers were delivering at home and the reasons were challenge in affixing their time to the appointment might deliver in the meantime. The problem is bolder in the distant places and not here since there Bajaj vehicle transport service to the HFs. Generally, in Tebelima Kebele, there is infrastructural challenge with road accessibility to get transport service. Those mothers in these areas, might hold back to come to the HFs from lack of support.

***Reasons for discontinuation across the continuum***

1. Why do women go to the facility for ANC, yet mostly deliver at home? Probe for; Financial barriers and opportunity costs, Distance and access, Socio-cultural, Quality of care and non-dignified care
2. In your opinion, what should be improved regarding facility delivery services? Continuity of care?

Respondent: I have nothing to say on the challenges and everything is great so far.

***Traditional practices during pregnancy, childbirth and postnatal period***

1. Can you tell us about the traditional practices and beliefs during pregnancy, delivery and postnatal period in your community?
2. Do you think these traditional beliefs, religious practices, and cultural norms affect mothers to use care during pregnancy, delivery, and postpartum period in your community? Explain how and why?

Respondent: No, there are no tradition or belief related factors hindering mothers to access the delivery services.

1. How do you see community volunteers/TBAs and health professionals and maternal health services provided to the community?

## Postpartum

***The practice of ANC, facility delivery, and PNC services***

1. How early do women go for PNC? **Probe** why do they go at that time? Why earlier or later?

Interviewer: Do women make follow up after giving birth?

Respondent: yes, they do after they gave birth and after 45 days for vaccination service for their child.

Interviewer: How about for their own advantage that is for PNC? And on which day do they start follow up after giving birth?

Respondent: They make their follow up for PNC after seven days of birth.

Interviewer: Do they seek medication?

Respondent: yes, they do.

1. How often do they go for PNC? **Probe** why do they go at that time?

Interviewer: Do postpartum mothers make follow up other that the seven you mentioned?

Respondent: she starts to make the PNC follow up starting from her 45^th^ days, and they might also visit before this day if they encounter any health problem. Otherwise, they only make the visit on the 45^th^ day scheduled for the newborn vaccination.

Interviewer: Why do you think is the reason for sticking to the 45^th^ day of vaccination for their PNC?

Respondent: pregnant mothers don’t think about the probability of encountering any health related risks. After the 45th days however, they make a visit to the health facilities thinking they might get pregnant after this day and to get a vaccination service for their babies as well. Mothers don’t believe postpartum mothers need PNC after their delivery.

Interviewer: Is there no any awareness creation made for the pregnant mothers on necessity of making PNC after delivery?

Respondent: there are no pregnant mothers who are giving at home, all have understood about the importance a facility delivery. During their delivery at HFs, they are educated to come on the 45^th^ day for vaccination purposes.

1. Do women think skilled attendance during postpartum helps their babies and themselves?

Respondent: it has a paramount importance, this is because, the mothers might be suffering from hypertension or may have contracted HIV and if she is not consulted at a HF level, she might be facing risks during her delivery.

Interviewer: What are the advantages of making a skilled attendance for PNC? And how long do they stay at the HCs after delivery?

Respondent: they stay at the HCs for 24 hrs. period until the mothers stabilizes and the providers follow here not the mothers to face similar challenges they would be facing in their homes, that is why it is required for them to give birth at the HCs.

***Reasons for use of ANC, facility delivery and PNC***

1. Explain factors that would motivate women to utilize PNC service in their pregnancy

Respondent: Well, it is for the sake of their baby’s welfare and themselves that they are visiting the HCs they are making the skilled PNC attendance.

Interviewer: What to do you think are the factors/reasons which made them realize this?

Respondent: Because a mother knows that is if she makes ANC follow up, beginning from early pregnancy she would deliver a healthy baby.

Interviewer: where did they get this understanding?

Respondent: well we discuss the issue among ourselves. Us the HDAs and the pregnant mothers takes health education when we meet every month concerning issues like the benefits of making ANC, and then PNCs and it will keep their baby’s healthy and other different issues, and this is what motivated them to know about the benefits of PNC starting from their early pregnancy.

Interviewer: What else have motivated them, any environmental reasons?

Respondent: Nothing else.

***Barriers for attending ANC, facility delivery and PNC use***

1. If women don't go for post-natal care, what are their reasons? What are barriers to accessing PNC? **Probe** for; Financial barriers and opportunity costs, Distance and access or lack of service, Socio-cultural, Quality of care

Interviewer: Are there any mothers who don’t come for PNC?

Respondent: No there is not.

Interviewer: Are you sure?

Respondent: Yes.

***Reasons for discontinuation across the continuum***

1. Why do women go to the delivery at the facility, yet mostly don’t receive PNC? Explain the obstacles influenced women to utilize skilled care during pregnancy, childbirth, and postpartum in your community? **Probe** for; Financial barriers and opportunity costs, Distance and access, Socio-cultural, Quality of care.
2. In your opinion, what should be improved regarding PNC services? Continuum of care?

***Traditional practices during pregnancy, childbirth and postnatal period***

1. Can you tell us about the traditional practices and beliefs during pregnancy, delivery and postnatal period in your community?
2. Do you think these traditional beliefs, religious practices, and cultural norms affect mothers to use care during pregnancy, delivery, and postpartum period in your community? Explain how and why?

Respondent: No, there are no tradition or belief related factors hindering mothers to access the PNC services.

1. How do you see community volunteers/TBAs and health professionals and maternal health services provided to the community?

# 5. IDI with recently delivered woman_Jabi_191102_1038

| **I** | **Section I: Identification** | |
| --- | --- | --- |
| 1 | Questionnaire ID | **191102_1038** |
| 2 | Area Identification |  |
| 3 | Name of Woreda | **Jabi; Maksegnit** |
| 4 | Name of Kebele | **____________________________________** |
| 5 | Name of moderator | **CB** |
| 6 | Name of note taker | **AA** |
| 7 | Date of discussion | **_______________________________** |
| 8 | Start time: | **______:________** |
| 9 | End time: | **20:46** |

| **II** | **Section II: Participant Demographic Intake Sheet** | | | | | |
| --- | --- | --- | --- | --- | --- | --- |
| 1 | Participant code |  |  |  |  |  |
| 2 | Age |  |  |  |  |  |
| 3 | Religion |  |  |  |  |  |
| 4 | Marital status |  |  |  |  |  |
| 5 | Are you employed? (Yes/No) |  |  |  |  |  |
| 6 | Educational level |  |  |  |  |  |

## Antepartum

***The practice of ANC, facility delivery, and PNC services***

1. How early do women go for ANC? **Probe** why do they go at that time? Why earlier or later?

Respondent: 3-4 months of pregnancy; there are some mothers who started early in 2 months

Interviewer: Why don’t they started early?

Respondent: we don’t know whether it is pregnancy or not. I knew in 2-3 months when I feel movement of the fetus. It is because we don’t know whether it is pregnancy or not.

Interviewer: are there mothers who started late like 6 months?

Respondent: No, they are not staying this long. I started at 4 months.

1. How often do they go to ANC? **Probe** why do they go at that time?
2. Do women think skilled attendance during pregnancy helps their pregnancy?

***Reasons for use of ANC, facility delivery and PNC***

1. Explain factors that would motivate women to utilize ANC service in their pregnancy

Interviewer: I see others are attending. HDAs are also mobilizing us. I also go when I get sick.

***Barriers for attending ANC, facility delivery and PNC use***

1. If women do not go for ANC, what are their reasons? What are barriers to accessing ANC? **Probe** for; Financial barriers and opportunity costs, Distance and access, Socio-cultural, Quality of care

Respondent: I don’t know. Nowadsys, they are following.

***Reasons for discontinuation across the continuum***

1. Why do women go to the facility for first ANC, but discontinue for subsequent ANC visits? **Probe** for; Financial barriers and opportunity costs, Distance and access, socio-cultural, Quality of care

**No one.**

1. In your opinion, what should be improved regarding ANC services?

None. They helped me well

***Traditional practices during pregnancy, childbirth and postnatal period***

1. Can you tell us about the traditional practices and beliefs during pregnancy, delivery and postnatal period in your community?
2. Do you think these traditional beliefs, religious practices, and cultural norms affect mothers to use care during pregnancy, delivery, and postpartum period in your community? Explain how and why?
3. How do you see community volunteers/TBAs and health professionals and maternal health services provided to the community?

## Intrapartum

***The practice of facility delivery***

1. Do women think skilled attendance during childbirth helps themselves and their babies?

Interviewer: How do mothers see give birth at health facility?

Respondent: it is important

Interviewer: what services did they give u?,

Respondent: they gave me glucose

***Reasons for use of facility delivery***

1. Explain factors that would motivate women to utilize delivery service in their pregnancy **Probe** for reasons for using continuum of care

They motivate us. They are mobilizing and teaching us to attend

***Barriers for attending ANC, facility delivery and PNC use***

1. If women deliver at home, what are their reasons? Explain the constraints that influenced women to utilize facility delivery services? **Probe** for; Financial barriers and opportunity costs, Distance and access, Socio-cultural, Quality of care and non-dignified care

Respondet: nowadsys, they are delivering at facility. They considered it as their mother’s home and stay there.

***Reasons for discontinuation across the continuum***

1. Why do women go to the facility for ANC, yet mostly deliver at home? Probe for; Financial barriers and opportunity costs, Distance and access, Socio-cultural, Quality of care and non-dignified care

Respondent: No one is delivering at home. They are going directly to facility and give birth there.

1. In your opinion, what should be improved regarding facility delivery services? Continuity of care?

Nothing. If they can’t; they refer us to Finteselam.

***Traditional practices during pregnancy, childbirth and postnatal period***

1. Can you tell us about the traditional practices and beliefs during pregnancy, delivery and postnatal period in your community?

Respondent: Nothing. We bath next day after delivery as they told us. Giving butter to the baby is now forbidden

1. Do you think these traditional beliefs, religious practices, and cultural norms affect mothers to use care during pregnancy, delivery, and postpartum period in your community? Explain how and why?
2. How do you see community volunteers/TBAs and health professionals and maternal health services provided to the community?
3. Explain the support you get from the community to and decision making on health services during pregnancy. delivery and postnatal period

They told us to go to facility and to attend

1. Explain us your experiences relating to the utilization of ANC, birth, and PNC care provided by skilled birth attendants. Prove for;

• their interactions with skilled birth attendants during ANC, delivery, and PNC

• their confidence in skilled birth attendants’ abilities, and

• respect and compassion of attendants ( respect for the traditional beliefs of the women, etc)

Respondent: When we went they accepted us well. They are nice [work nachew]. They are not happy when women give birth at home. [bet siwoldu new yemitenawotu]. They would be angery when delivered at home. Otherwise, they are very nice in all aspects. [hulemenachew shega nachew]

## Postpartum

***The practice of ANC, facility delivery, and PNC services***

1. How early do women go for PNC? **Probe** why do they go at that time? Why earlier or later?

Interviewer: Do women go to facility for PNC?

Respondent: Ya, they are following.

Interviewer: u told me u delivered at HC. Do u attend PNC?

Respondent: I just recently delivered. I followed monthly during pregnancy.

Interviewer: Did they tell u to come back for PNC?

Respondent: they told me to get back on 7^th^ (after 24 days after delivery) [looks for vaccination], but I did not go because there was someone’s mourning ceremony.

Interviewer: Why don’t u go till today?

Respondent: I missed the schedule. Then after that I get busy for his baptism ceremony. It is tomorrow.

1. How often do they go for PNC? **Probe** why do they go at that time?
2. Do women think skilled attendance during postpartum helps their babies and themselves?

Respondent: ya, they thought it is important. Is it about FP?

Interviewer: it is not clear for me.

***Reasons for use of ANC, facility delivery and PNC***

1. Explain factors that would motivate women to utilize PNC service in their pregnancy

***Barriers for attending ANC, facility delivery and PNC use***

1. If women don't go for post-natal care, what are their reasons? What are barriers to accessing PNC? **Probe** for; Financial barriers and opportunity costs, Distance and access or lack of service, Socio-cultural, Quality of care

***Reasons for discontinuation across the continuum***

1. Why do women go to the delivery at the facility, yet mostly don’t receive PNC? Explain the obstacles influenced women to utilize skilled care during pregnancy, childbirth, and postpartum in your community? **Probe** for; financial barriers and opportunity costs, Distance and access, Socio-cultural, Quality of care.
2. In your opinion, what should be improved regarding PNC services? Continuum of care?

***Traditional practices during pregnancy, childbirth and postnatal period***

1. Can you tell us about the traditional practices and beliefs during pregnancy, delivery and postnatal period in your community?
2. Do you think these traditional beliefs, religious practices, and cultural norms affect mothers to use care during pregnancy, delivery, and postpartum period in your community? Explain how and why?

Respondent: No, there are no tradition or belief related factors hindering mothers to access the PNC services.

1. How do you see community volunteers/TBAs and health professionals and maternal health services provided to the community?
