# Supplementary material for: Community’s experience and perceptions of maternal health services across the continuum of care in Ethiopia: A qualitative study
Source: PLoS One. 2021 Aug 4;16(8):e0255404. doi: 10.1371/journal.pone.0255404 (PMC8336848; doi:10.1371/journal.pone.0255404)
Supplement: S4 Appendix — These are focus group discussion transcripts used for this analysis. (DOCX) [file pone.0255404.s004.docx]

FGD Transcripts

# FGD with community leaders_Girarima_Quarit_191105_0131

| **I** | **Section I: Identification** | |
| --- | --- | --- |
| 1 | Questionnaire ID | **01** |
| 2 | Area Identification | Elder group (72, 42, 41, 67, 50, 52, 50 and 41 years old) |
| 3 | Name of Woreda | Quarit |
| 4 | Name of Kebele | Girarima |
| 5 | Name of moderator | CB |
| 6 | Name of note taker | AA |
| 7 | Date of discussion | 5-Nov-19 |
| 8 | Start time: | **______:________** |
| 9 | End time: | **____:______** |

| **II** | **Section II: Participant Demographic Intake Sheet** | | | | | |
| --- | --- | --- | --- | --- | --- | --- |
| 1 | Participant code |  |  |  |  |  |
| 2 | Age |  |  |  |  |  |
| 3 | Religion |  |  |  |  |  |
| 4 | Marital status |  |  |  |  |  |
| 5 | Are you employed? (Yes/No) |  |  |  |  |  |
| 6 | Educational level |  |  |  |  |  |

***The practice of ANC, facility delivery, and PNC services***

1. How early do women go for ANC? How early do women go for PNC? Why do they go at that time? Why earlier or later?

R1: when women became amnohorric for more than a month they will have nausea, fatigue, vomiting and poor appetite. This time she will be confused about their pregnancy status and the community tell her that "check your pregnancy status at health center" based on this they start pregnancy follow up.

On the other hand, when the women reached at 3rd month pregnancy they fill fetal moment and this time they go to pregnancy follow up

R2: Pregnancy is known at 3rd month, pregnancy related sign and symptoms less than three months considered as any other illness. At third month of the pregnancy majority of the pregnant women become fatigue and ill due to this they go to pregnancy screening and start pregnancy follow up. If they are healthy at third month of the pregnancy, they will start ANC lately because we are farmers and women become too busy for daily activities. Once they screened, they start ANC in the health center. if the pregnancy is normal they proceed ANC in the health center and if the HC staff consider the pregnancy isn't normal just they refer to higher level facilities for better follow up.

1. What kinds of services do they receive in ANC, childbirth and PNC? Are they satisfied?

Summary: They provide delivery service, drugs which are important for infant development and see the fetal presentation. Previously they were providing clothe and soap for the newborn but now they have stopped, just it was stimulation for facility delivery and pregnancy follow up. Health centers were supposed to provide transportation and coffee ceremony (coffee & porridge) after delivery for women but they are not doing it. We don't know the exact reason the ambulance is serving them (to mean the health providers) and there is no coffee ceremony. The community is raising fund for facility coffee ceremony yearly based, but the women aren't getting the service and it is discouraging for us. In general, the service isn't going well, even their approach isn't good.

R1: The health center staff approach and knowledge isn't good at all. I had taken my wife which were on labor and duty health provider shouted at me that "she is not term and on labor" and pushed me out. Then after I was waiting outside far from delivery room for a while and later on he called and invited me to come to delivery room since she delivered. I was not happy to get back since he forced me to send out, finally I had forgotten and came to the delivery room. By chance the new born was too small and the health provider told us that " I don't give immunization for the newborn since it is too small and it will not survive any more". We begged him for the new born immunization but he didn't change his mind and send us back home. The newborn took immunization at the health post and grew well and it is funny for my family now.

1. Do women think skilled attendance during pregnancy, childbirth, and PNC helps their pregnancy, babies and themselves?

R1: Previously delivery was at home and there were multiple challenges, now facility delivery solved many things. Example my wife delivered at home her first pregnancy. This is because she don't like going to health facility with fair of chemical injection (merz merfie)/family planning which make them in fertile. During home delivery the fetus died within the uterus and then we took her to the facility lately and she was in critical condition at that time. When she gets pregnant for the second time, she started follow up early and the health center referred her to the hospital and had follow up four times. Finally, on the onset of labor we went to the health center soon and the HC referred us to hospital since the presentation was not ok. The ambulance driver switched of his phone for HC staff and us. We reported it to the woreda officials and they let the driver come to us soon, but the travel was not safe since the driver stimulated with chat and there was conflict between the driver and us. Finally, she delivered in the hospital and the newborn was admitted for a month, if she were at home she would die, so facility delivery is important.

R2: My wife doesn’t like the service provided at the health center based on the rumor (merz merfie/family planning injection which make infertile) which where was spoken in the village and she gave birth three times at home. When she gave birth for third time the placenta retained for two days and she was bleeding a lot and in critical condition. When she gets pregnant for forth time she had follow up at the health center. Labor started at night, we couldn't find ambulance and finally we tried to take her in person and she delivered on the way. Even though she had delivered on the way we took her with the newborn for placenta removal and the health center staff shouted at me for the delay. They removed the placenta soon safely. Even though we couldn't reach on time at the health center for delivery they helped us for placenta removal. The facility staff teach us about maternity waiting home and the benefit of facility delivery, but since we are busy for our livelihood activity we couldn't do it. In general facility delivery is good.

Summary: Moderators proved that, "do you think that health facilities give merz merfie (family planning injection which make infertile)"? Some participants said that "it is rumor, government bring good things for us and we have never seen any incidence still now". One respondent also said that "previously there was a rumor the politicians give merz merfie (to mean acid/chemical injection) for their opposition groups, but no one give it to women just it is rumor"

***Reasons for use of ANC, facility delivery and PNC***

1. Explain factors that would motivate women to utilize ANC service in their pregnancy

Respondent: Health post staff (HEWs) motivate pregnant women to attend ANC and diliver at health center. In addition to this health insurance motivates the community and the follow up is important and people learn each other.

***Barriers for attending ANC, facility delivery and PNC use***

1. If women do not go for ANC, what are their reasons? What are barriers to accessing ANC? **Probe** for; Financial barriers and opportunity costs, Distance and access, Socio-cultural, Quality of care

Summary: The following are the possible challenges to attend ANC, delivery and PNC.

1. The river in between the kebele and the health center is the biggest challenge for us. Impossible to cross the river from June to September and this time no one has access for service which was provided at the health centres. Previously there was temporary bridge, but it was damaged by seasonal feeding due to heavy rain in the area. After that pregnant women are suffering a lot. Example in the last rainy season (June to September/2019) four labouring women died due to the river.

2. Shortage of health facility which give delivery service in the kebele.

3. Quality of care given in the health center is poor and there is partiality to give service. They have special attention for the clients they know/when the client has a care taker who is social for facility staff. one respondent said that "I took my severely ill wife for placenta removal, just we took her in shock position (we learned from the health professionals) by traditional streture which was carried by a person (qareza). The health provider had examined her and told me to take her back home since she is already died, if I try to remove the placenta things will be aggravated so it better if you take home or hospital". The time was mid night and just I gave up and didn't try to take her to hospital. We were not giving her anything even water until we reach the HC. After the provider told me to take her back home, I stated providing home remedies like sugar with water, rise, butter .... in the HC and get improved. When the health providers come to her in the early morning she was improving and they asked me the reason why? And I told them just things I did. Then they removed the placenta and asked me fee for the service they provided. Moderators proved that, do you pay for services given at the health center? of course it is free but placenta removal was done by health provider working in the private clinic, the payment is for him. So there is no well trained staff at the health center.

4. Ambulance service is almost nil and this is a challenge for us. Just we carry the labouring women to the bus stop but we don't get ambulance soon. Rarely when wet ambulance the drivers are crazy and they drive faster and it is another pain for women. The other problem is the ambulance isn't giving round trip service and we have no any other means of transportation mechanism to get back home and this a big challenge for us. Specially some community members are not taking critically ill women (preganant or labouring), because if she dies in the health center or hospital returning the dead body will be too expensive (3000-4000 bir/trip).

5. Women have no time for facility visit since they are too busy at home, especially when there are small kids at home.

6. There is no road access in all-weather condition. Example four patients has been taken to the HC by kareza in the morning.

7. Shortage of many for outside prescriptions and refreshment for people caring the kareza round the trip (since there is no ambulance service)

***Reasons for discontinuation across the continuum***

1. a)     Why do women go to the facility for first ANC, but discontinue for subsequent ANC visits? Are all mothers abiding to their appointment dates for ANC consultation?

b)    Why do women go to the facility for ANC, yet mostly deliver at home?

c)     Why do women go to the delivery at the facility, yet mostly don’t receive PNC? Are all mothers abiding to their appointment dates for PNC consultation?

Probe for financial barriers and opportunity costs, geographic barriers, socio-cultural barriers and quality of care barriers

Summary: The following are the possible challenges to discontinue ANC, delivery and PNC.

1. The river in between the kebele and the health center is the biggest challenge for us.

2. Shortage of health facility which give delivery service in the kebele.

3. Quality of care given in the health center is poor and there is partiality to give service.

4. Ambulance service is almost nil and this is a challenge for us.

5. Women have no time for facility visit since they are too busy at home, specially when there are small kids at home.

6. There is no road access in all-weather condition. Example four patients has been taken to the HC by kareza in the morning.

7. Shortage of many for outside prescriptions and refreshment for people carrying the kareza road the trip (since there is no ambulance service)

1. What costs do you think involved for pregnant women to attend ANC, facility delivery and PNC services?

Summary: The followings are costs for pregnant women:

1. Refreshment fee (round the trip) for people carrying the kareza (off road)

2. Outside prescriptions. Moderator probed, reimbursement for outside prescription fee and they responded that, "reimbursement is in the woreda, not in the health center and it is too far from the HC which is too difficult for us".

3. Transportation fee to returning back for on road

4. Yearly contribution for maternity waiting home.

8. In your opinion, what should be improved regarding ANC, facility delivery, and PNC

1. Knowledgeable/skilled health professionals are needed in the health post and health center example doctors in the health center and nurses in the health post are needed

2. Some facility staff behavior/ approach to the clients should be corrected because they are ignorant

3. We need road access and bridge to cross the river

4. Transportation mechanism should be corrected

5. Medical instruments/like ultrasound are needed in the health center

***Traditional practices during pregnancy, childbirth and postnatal period***

1. Can you tell us about the traditional practices and beliefs during pregnancy, delivery and postnatal period in your community?

Respondent: Previously we used to traditional drug to stop postpartum bleeding and addition to this we use religious practice (reading melke rufael) to stop bleeding. There is no any other traditional practice now a day.

1. Do you think these traditional beliefs, religious practices, and cultural norms affect mothers to use care during pregnancy, delivery, and postpartum period in your community? Explain how and why?

Respondent: Reading Melke rufael is important but everyone has no access for priest

1. How do you see community volunteers/TBAs and health professionals and maternal health services provided to the community?

Respondent: Health extension workers are working hard to send women for ANC and delivery going home to home and the community appreciate this. Some staff members in the health center also are supportive but some others have no good approach and they are not knowledgeable to give the service.

***For community and religious leaders and community volunteers only***

***Community perceptions about health providers and maternal health programs***

1. How the community see the maternal health programs and health professionals? Tell me the perception about maternal health care services. Perception about different care providers.

Mentioned in the above responses

1. What efforts your community made to increase maternal health service in your community?

The community members are contributing year contribution for maternity waiting home and carry labouring mother to the health center and vis versa.

# FGD with HDAs_Tiatia_Burie_191103_0440

| **I** | **Section I: Identification** | |
| --- | --- | --- |
| 1 | Questionnaire ID | **02** |
| 2 | Area Identification | **Tiatia** |
| 3 | Name of Woreda | **Bure** |
| 4 | Name of Kebele | **Tiatia** |
| 5 | Name of moderator | **CB** |
| 6 | Name of note taker | **AA** |
| 7 | Date of discussion | **12/23/2019** |
| 8 | Start time: | **______:________** |
| 9 | End time: | **1:39** |

| **II** | **Section II: Participant Demographic Intake Sheet** | | | | | | | | | | |
| --- | --- | --- | --- | --- | --- | --- | --- | --- | --- | --- | --- |
| 1 | Participant code | P1 | P2 | P3 | P4 | P5 | P6 | - | - | - | - |
| 2 | Age | 45 | - | 42 | - | 55 | - | - | - | - | - |
| 3 | Religion | - | - | - | - | - | - | - | - | - | - |
| 4 | Marital status | - | - | - | - | - | - | - | - | - | - |
| 5 | Are you employed? (Yes/No) | - | - | - | - | - | - | - | - | - | - |
| 6 | Duration of employment | 6 yrs | 6 yrs | - | 13 yrs | 6yrs | 1 yr | - | - | - | - |
| 7 | Educational level | 0 | 0 | 0 | 0 | 0 | 10 | - | - | - | - |

P1- Workenesh Niguse, P2- Musit Bekele, P3-Bitew Kasahun, P4- Enat Tadesse, P5-Shitaye Gendebew, P6-Abeba Tafere

1. **Practice of ANC, facility delivery, and PNC services**
2. How early do women go for ANC? How early do women go for PNC? Why do they go at that time? Why earlier or later?

Moderator: How early do women start ANC after their pregnancy in this locality?

Participant response: the recommendation is to start within three months, however, they usually start earlier than that, because, women notice absence of their period starting from month one, and if it doesn’t appear in the second month they intend to be tested for pregnancy and if they are found pregnant, they will be told to start their ANC at the HFs.

Moderator: But when do they normally start their ANC?

PR: They start in their second month of pregnancy.

Moderator: Do all pregnant mothers under each of you HDAs, always start their ANC in the second month?

PR: there are some women such as students who starts timely when they don’t see their period. However, those women who are farmers, we identify them when their pregnancy is physically noticeable and we advice them to go to the HFs and get tested. If she is found pregnant, the health care provider might supply her with drugs to take in orally and appoint her. So there are who starts early within two months, three months and late on the fifth month.

Moderator: What is the reason behind for some to start early within two months and some late after three months?

Participant response: It is the lack of education by women. If I am educated for instance, and if expect that my period comes this month and doesn’t appear, and similar for the second month, then I will go and get tested. However, for the uneducated mothers, it is from the noticeable increase in size of their uterus that they assume they might be pregnant but they might get pregnant without seeing menstruation; for those who have been using the injectable family planning method might not see menstruation instantly and usually see sometime after its removal, however, they should be tested for pregnancy, because they might be found pregnant in the mean time. Anyhow, it is this assumption of delay in menstrual blood usually after removal of the injectables that is causing delays in making ANC if they are found pregnant.

Moderator: can we say that not seeing menstruation by women could be misled by the use of family planning, caused due to their long use of family planning? And could family planning be one of the reason to delay ANC, misleading absence of menstruation complicated from long use of family planning?

Participant response: yes it could be a reason. Women might get comfortable with the type of family planning methods they have been using, be it injectable or implant, and if they are comfortable with the method, their period might not appear after its removal. The injectable for instance might cause absence of menstrual blood for two or three months, and this might misled them not to go and tested for pregnancy and this might be the reason for women not start their ANC early.

Moderator: What other reason is there, for instance are there women who knows their pregnancy and deliberately delays or don’t start ANC?

Participant Response: No, there are not. Previously, before the introduction of the ANC service, women used to get pregnant and delivers in their homes without making any ANC follow; bearing in mind also those who dies and survive by chance in the process. However, after the introduction of the ANC service and the education that we got from the HEWs, we are able to identify women who are out of their normal menstrual cycle, through friendly inquiries to know the status with the woman’s menstrual normal cycle and encourage those who didn’t see their period for more than two months. This is different for instance in those who are educated, who notice early within two or three days of absence in their menstrual blood and go and tested for pregnancy. There are also those we educate after their pregnancy is confirmed to follow their schedule for ANC at the HFs.

Moderator: Ok we will come to that later but is there any other reason for some women for not starting their ANC early?

Participant response: for instance, there was a women in our locality, who is young, and it was her second pregnancy, and this women didn’t know she was pregnant for the same reason thinking her period didn’t come because of the injectables she has been using, she didn’t realize it until the fetus starts moving at the fourth or fifth month of pregnancy and which delayed her ANC follow up and a little later her labor came. I was accidentally there at the HC when she came to deliver her baby and she was told by the HC that it was not possible to deliver there since she was not attending her ANC in the HC. After some pleading, they accepted her and informed her she is left with one month from her due date but she delivered on the same day. What made this gap is the lack of awareness and mis-perceived the absence of menstrual blood for complication resulted from the injectables family planning method she has been using and also the fact that she is young, which would have been different if she was a grown up women which she would have identified her status with pregnancy.

Moderator: what else?

Participant response: Not now in the current time, there is not much women who doesn’t follow their ANC, they start their ANC as soon as they realize their status, however, in earlier times, they used to even swear at us when we ask them to go for ANC, they used to question our presence as bad spirited which was not different for all ages of women. But now there is no one questioning of our presence and they attend ANC eagerly and interested.

Moderator: Do you mind explaining the word you just used ‘SHAKLEW NEW YIMIHEDU’?

Participant response: [laughter…], it is to mean they tend to make the ANC visit with interest, its our local wording (language).

Moderator: How early do women go for PNC?

Participant response: they make their PNC after their 45 days.

Moderator: Don’t women go to the HC if they encounter any illness or bleeding before this day related with her delivery?

Participant response: yes she will go to the HC and the health care providers won’t let her go if they see her bleeding, and might spend her night or day there until she recovers, and go back again on the 45^th^ day. So women make a visit to the HC before the 45^th^ day if she is sick otherwise, if she is apparently healthy, she will visit the HC at the 45^th^ day of delivery. However, for those who are educated make a return for check up on the second day after her release from the HC and if she is healthy, she returns on the 45^th^ day.

Moderator: Do all women make the same visit on the second day after delivery for check up?

Participant response: other than those who are living far from the HC, the rest women under our watch make the check up on the second day. There are three Kebeles which are far and Ambulances are called for these Kebeles. Other wise the rest under our watch makes a swift check up to the HC and they don’t interrupt.

Moderator: Do the HEWs assigned in these Kebeles make a home to home visit and check postpartum mothers after delivery?

Participant response: yes they do. They go to postpartum homes on the third day, it is if the HEWs don’t make these check up that postpartum mothers go for check to the HCs by themselves. It is when the HEWs allows it that the postpartum mothers visit the HC for third or second day check up.

Moderator: Is it made for all postpartum mothers by HEWs?

PR: Yes for women found in the Kebeles they are assigned, there are mechanisms the HEWs are notified of the births in their Kebeles through a written note from us and the HEWs read the note and make the visit to every home there is birth.

Moderator: How long do the HEWs wait in days to make the home visit after the postpartum delivery?

PR: on the same day they received the birth notification note. They don’t spend a day in their health post without asking about such birth notifications. It is us the HDAs that brings the birth notification paper which we bring to the HP on the same day, we also get notified from her husband.

1. What kinds of services do they receive in ANC, childbirth and PNC? Are they satisfied?

PR: they provide her an iron tablet and also provide her with supplements for nourishing the fetus. If she is healthy, they advice her on locally available food that the pregnant mother should be feeding herself in her home and they will also vaccinate them with Meningitis or Tetanus vaccines.

Moderator: You meet almost all women under you and you have the chance to understood their feelings, are they satisfied with the services provided to them by the HCs?

PR: there are appointments made by the registration or labor and delivery unit, asking the mothers in the afternoon and the next day at the HC, which is challenging for the mothers.

Moderator: What else?

PR: they pregnant mothers have a place, mothers waiting room, they use to stay at the the HC when their labour nears.

Moderator: Yes I know, but what other complains do the pregnant mothers have about the services at the HC?

PR: there are none other the previous one mentioned.

Moderator: Any other if you have?

PR: I have not heard any other problem so far.

Moderator: What supports and services are provided for pregnant women by the HC when women come for delivery?

PR: the HC prepare the postpartum woman a porridge and coffee and, if she is going to spend the night, it will serve her with dinner and breakfast and lunch as well.

Moderator: How long do the postpartum women stay at the HC after delivery?

PR: They stay for 24hrs long. They spend the night and day, and leave.

Moderator: Is there anything that needs improving during delivery, which they suggested? Are they happy?

PR: it is the lack of competent midwife in the HC for pregnant mothers in our locality. This would have prevented pregnant women from suffering when they are referred to other HCs from the Ambulance’s bouncy driving on the rough roads and from their labor. Once there was a pregnant women who spend the whole day and finally referred by the HC to other HF, but it would have been better if there was competent midwives here than send her to other place, and she would not have suffered. The HC is located in a suitable location but there was a problem last week when the parents asked the HCs to refer them and they kept the women longer and finally referred her but the pregnant mother didn’t make it. It was not necessary for them to keep the pregnant women that long if they were unable to deliver her.

Participant response: there is a problem in this HC like recently there was a challenge in letting the pregnant women enter the HC during labor, and no food or coffee were provided to the mother after her delivery. And during her labor, there was also a delay and when we ask him if he is waiting until she dies, he told us to wait with arrogance, and the mother in labor was also very uncomfortable and told us to say nothing, finally she was able to deliver with the help St. Mary and to make it home.

Participant response: there is no porridge or coffee provided to the mother, we want to ask what is causing them unable to make this supports, is it because they are not having adequate support from the government and why are they just letting us leave the HC in hurry, involving the guards, I even provided my comments on this issue this year. While the women I mentioned earlier came for labor once in this HC, the female guard separated the pregnant mother from her daughter who came along with us to the HC, slammed the door on her and, only the mother and I spend the night alone in the separate room. The female guard also once mentioned why I entered the room with my feet soiled and make the floor dirty, which I replied to her that she would have been shitting (be diarrheic) if she were to walk all the difficult road to here like me instead of standing here. She refused to open the doors and which I opened forcibly and presented my complains on her treatment to the HC.

Participant response: About the porridge, the Kebele identify and provides the means. The Kebele provides the land which is used to purchase the porridge supplements, however, the Kebele is not providing the porridge for three years and now we are on the fourth year and the Kebele is not providing a land for this purpose and the pregnant women which are in this Kebeles are not provided with the porridge at the HC. This is making impartiality among the pregnant women during their labor.

Moderator: Are there women who refused to come to deliver at the HC from the non-contributing Kebeles, because they are not getting support from the HC?

Participant response: No we didn’t encounter women refusing to come for this reason, and even if the women don’t get it from the HC, porridge and coffee are provided from the community or their family, but the HC when asked for the materials to make the porridge and coffee for the postpartum mother from one of the the non-contributing, they are not willing and would say the store used for putting these materials is locked.

Participant response: the other gap is in the provision of cloth for the postpartum mother and newborn after birth, however, it is not the practice any more and mothers were complaining why the provision of these clothes has stopped from the HC.

Participant response: it was for the newborn not for the mother that towels and soap were provided from supporting NGOs and now the NGO is more active, the complain was on why this has stopped from the NGOs.

1. Do women think skilled attendance during pregnancy, childbirth, and PNC helps their pregnancy, babies and themselves?

Moderator: How do the women in these locality see the benefit of the skilled delivery service?

Participant response: They don’t have a limit to how they are satisfied because now there is no risk to the mother or the newborn during delivery at the HCs and also free of measles disease and the newborn come out healthy.

Participant response: we used to give different supplements to the mother in earlier times to ease up the labor for the mother, however, now there is better conditions with an easy labor when they deliver at the HCs and the community has a positive attitude towards the services provided.

Moderator: What other comments do the community have on the delivery services or the ANC or PNC services?

Participant response: the services have benefits and there are no much who refuse to go for these services.

1. **Reasons for use of ANC, facility delivery and PNC**
2. Explain factors that would motivate women to utilize ANC service, institutional delivery, and PNC, **Probe** for reasons for using continuum of care

Participant response: the WDAs teach us on maternal care and also the observation of the community and encouraged when they see pregnant women return delivering safely from HCs. Women are encouraged to make ANC visits when they others following ANC and being healthy doing so. Previously delivery was dreadful thing and there was local ways of stopping bleeding which is not successful but now days it is not so a risk and no one worries about a pregnant women dying of delivery after the establishment of the HC in the locality.

1. **Barriers for attending ANC, facility delivery and PNC use**
2. If women do not go for ANC, delivery, and PNC what are their reasons? Explain the obstacles influenced women to utilize ANC, PNC, facility delivery services in your community? Obstacles using different care providers? **Probe** for financial barriers and opportunity costs, geographic barriers, socio-cultural barriers and quality of care barriers.

Participant response: they re hold back from their previous beliefs and they also respond to the HDAs during their visits that they re there for their own benefits or perdiem not that they cared about the pregnant mothers well being.

Moderatort: What else is there they bring up as a reason for their lack of interest?

Participant response: they also mention they get shy showing of their naked body during examination and they have also fears of being touched and hands of health professionals inserted in to their vagina for examination. This is because some don’t have the awareness otherwise, there are many women who are being examined the same way.

Participant response: this was true in the previous times, I myself got the same experience while I was pregnant. I felt some pain when I was four month pregnant, and they took me to a health facility and the health professional did just hit me with a fist on my belly without examining me first, it was why I named the boy ‘Girum’ after, the fetus reacted by moving inside of me when he hit me on my belly. He then told me the fetus is dead inside of me and told him what kind of a doctor he is and as I was trying to go out he pulled me inside, repeated the same fist and locked me inside the room and leave. I started to panic and wondered what was going to happen that day. He came back after sometime swear at me as unmannered and then he called my husband and insulted me as I am ‘Donkey like’ to my husband and told him that I was not going to survive that night, and my family got scared and took me out and later I was able to deliver safely and named the child ‘Girum’. and it is to mean I know that this things has happened in the past.

Moderator: Any other reason for instance you told us, they believe in the mercy of the Virgin St.Mary to safely deliver or distance, is it not a barrier?

Participant response: if the village is far, the community will bring her with a locally made carrier and those near to the HC will be transported through an ambulance.

Moderator: Are there any women who doesn't come to deliver because of the ambulance challenge?

Participant response: No they don’t interrupt or preferred not to come to the HFs for this reasons.

Moderator: Are there any women who dis-continue ANC after they start?

Participant response: there might be some who discontinue but it depends on the locality. Women would come when they have complains, take medications and if they were appointment after a month time, they forget it because they are healed and have no more complains and might come when they have complains again and not on the day of their appointment.

Participant response: if they start ANC here, the professionals appoints them and come back on the day of their appointment.

Moderator: Is it when they are sick or because they are pregnant that women start to make their ANC?

Participant response: Aha, this was what we have been telling this whole time, we said they start their ANC from day one.

1. **Reasons for discontinuation**
2. Why do women go to the facility for first ANC, but discontinue for subsequent ANC visits? Are all mothers abiding to their appointment dates for ANC consultation?

Moderator: Are there any women who have been following their ANC but don’t go to the HF when it it time to deliver?

Participant response: No there are not, not in our locality, as far as our Kebele is concerned and this is not above their well being. We only know what is in our kebele and not regarding the situation with the other kebeles.

Participant response: the delivery couch that is available in the HC is not wide and is very narrow and not very comfortable. The couch is not suitable for delivery purpose. When we ask them to be with the pregnant women during her labour, they won’t allow us and no none is there along side the women, and they are supporting us in this regard and it is not right to prevent us from entering the labor ward.

1. Why do women go to the facility for ANC, yet mostly deliver at home?
2. Why do women go to the delivery at the facility, yet mostly don’t receive PNC? Are all mothers abiding to their appointment dates for PNC consultation? **Probe** for financial barriers and opportunity costs, geographic barriers, socio-cultural barriers and quality of care barrier

Moderator: Are there women who delivers at the HC but don’t return back on the next two or three days for check up for PNC?

Participant response: yes there are who doesn't come and if she is sick they don’t come back. The reason they don’t come back on the second is if she doesn’t see any discharges after her birth which means she is apparently healthy.

1. What costs do you think involved for pregnant women to attend ANC, facility delivery and PNC services?

Moderator: Are there any expenses made for services (delivery, ANC or PNC) provided to pregnant mother?

Participants response: there are no payments, if there are even costs enquire from drugs bought outside of the HC, it will be reimbursed from the health insurance system.

Moderator: Would the ambulance that transported the mother to the HC return her back after delivery?

Participant response: we have told you earlier, the ambulance doesn’t return her back, she will return using her own transportation mechanisms and yes there expenses that the mother spends for transportation service for returning back.

Moderator: What else, any other cost?

Participant response: we spend costs for purchasing bottled water.

Moderator: How many people would accompany a mother when she comes for delivery?

Participant response: A lot of people would come, and mostly sisters or brothers would want to enter the labor ward, initially also during early stage of the labor it would be allowed for most people to be within the same room with the pregnant mother. During late stage of the labor or when she is taken to delivery room, one or two people would be allowed to enter, *[a lot of objection from the other participants were heard as this participant was explaining this],* but my mother were allowed to enter after I delivered. Up to two people will also be allowed to enter the ambulance during transport of the mother to the HC during labor, the rest who accompany her will have to use their transport from their own pockets and also the women covers her own cost for transportation for returning back home after delivery.

1. In your opinion, what should be improved regarding ANC, facility delivery, and PNC?

Participant response: I would recommend if the HC doesn’t discontinue the provision of the clothes to the newborn after delivery, that was once the trend. This is as far as the HC gets support to provide the means.

Participant response: I otherwise recommend for the ambulance to return the postpartum back to her home in place of the provision of clothes to the newborn.

Moderator: what else do you need to improve?

Participant response: It would nice for the health professionals not to insult the mothers while they are on labor. The other improvement needed is for a competent midwife to be availed to avoid the referral to other HFs. Improvement also needed on the supply of nutrient to the mother, there a push and follow up for the Kebele to allocate a dedicated land that will be used for supplying of food for the pregnant mothers during their delivery, the authority need to stop being ignorant on the kebeles.

Participant response: the other challenge the health professionals are usually playing a card while the pregnant is screaming and suffering from the labor and while her status should have been monitored and when we provide them feedback and to even refer her to other HFs if they can not do anything about her, they would get belligerent and tell us to leave and take her out if we want. No one would know if she is bleeding or had lost fluid (amniotic fluid early) if they don’t monitor her status regularly.

Participant response: for instance, there was one HDA of our own, and she went to the HC for delivery at night time. It is known that when we loose our amniotic fluid means the baby has arrived, and there was no one when this happened to her in the HC and when she instructed her husband to help her stand, the baby was deliver and still no one was there to help them and this needs to be corrected.

Moderator: is there any other service you think should improved?

Participant response:

1. **Traditional practices during pregnancy, childbirth and postnatal period**
2. Can you tell us about the traditional practices and beliefs during pregnancy, delivery and postnatal period in your community?

Moderator: Now you can tell us about the traditional practices that are common done during delivery or postnatal periods in this community?

Participant response: Eree, unless it is for previous times, there are no such things in the current time.

Participant response: now in the current in the ANC era, there will be feast that will be made on to respect the occasion of birth. Relatives will come to her to nourish her and she will be nourished well and she will be prevented from having sexual intercourse and would take FP during the 45^th^ day vaccination.

Moderator: for instance, in my village there is a trend of providing the newborn with raw butter to prevent him from constipation.

Participant response: for instance, I can tell you that I attended 17 births, and we suggested to make the newborn taste butter and we also instruct the mother to wash her breast and discard the first part of the breast milk and feed the newborn after. Nowadays, we are aware the first part of the breast milk is the most important and we don’t do that anymore.

Moderator: How about making gun fires with the belief to scare way ‘Satan’ from the postpartum mother?

Participant response: those things were from previous times; we used to create noise from tray nickels used for toasting cereals, and making gun fire near the mothers ear were those in our old times. It is no more the practice in the current time.

Moderator: Are there things that traditionally prevents the pregnant mother from engaging because of her status?

Participant response: pregnant women were prevented from working in earlier times but now she is engaged in her chores and delivers when her time comes to deliver.

1. Do you think these traditional beliefs, religious practices, and cultural norms affect mothers to use care during pregnancy, delivery, and postpartum period in your community? Explain how and why?
2. How do you see community volunteers/TBAs, health professionals and maternal health services provided to the community?

Moderator: how do the community see the services provided to pregnant mother by the health professionals?

Participant response: we the HDAs are working as volunteers, leaving our home, house hold responsibilities, our goats, cattle which should have been under our watch behind, but the community see us differently as getting benefits and that we are working for the sake of getting perdiems and compensated where actually we are not getting anything.

Participant response: the HC health professionals are obedient and willing to serve the community. The woman assigned in the registration unit works forcibly by begging. For instance, I have my health insurance but might not have my card with me and when we ask her to give us our registration card she tells us we have to bring our card first; this is not possible to do rather they should have write the card number at the back of our health insurance book other wise it it possible since the registration card is easily lost.

Participant response: the other is on the HC’s guards; they say only two persons who are carrying patients are allowed to enter the compound, however, some people who are accompanying the patient might be drunk and may get in conflict with the guards and the community complains about the service.

Participant response: the community generalizes the complains from the guards on the HC service as not making any changes nor having a competent professionals and would reflects on betterment of the HC being closed rather than continuing its existence. There is a saying that ‘the first is better than the second’, there was a woman once who was not approved that she married a short man and then she married a longer man but accompanied with his dog, and the community coined the saying , ‘Anchi melke Ayalew Zenderos Letenkua

Bamarerew Base Esteweshaw Meta’. the reason why I mentioning this is that we were complaining of the past but if there is serious assessment of the current situation there is more to complain. The community is commenting on the currently assigned health professionals, as what kind of professionals has been assigned saying that they can’t provide them with registration replacement, and I hear the community complaining about the newly and appointed professionals and I am using the previous saying as to give them some time and that they might be better if they are given time.

Participant response: there are no other service related challenges in the HC, however, the health professionals don’t provide service in Saturdays unless the patient is a child, however, any adult can get sick in Saturdays and might need medication, they say that they don’t treat adults unless for head injuries and emergencies, and sometime, they also provide service through payments. ***[other participant also objected her on service not being given on weekends by the HC, some say over spoke as it is not always true], the participant continued after as,*** this is my experience of the HC.

Participant response: they have started providing the service even in Sundays.

Participant response: I also accompanied one person for Tetanus vaccine and they are providing service on Sundays.

Participant response: No it is not good to comment them like that because they are providing service on Sundays as well. They provide service for pregnant women coming for delivery but we can comment the treatment of the female guard who doesn’t the door, and I even told the ambulance driver not to take me early before the female guard’s face and I even told her that I will not leave the HC unless I see her face. But the ambulance took early in the night.

Moderator: this is the last one, how is the community contributing to facilitate the smooth provision of health care service to pregnant mothers?

Participant response: the community participates in digging of wells for latrine construction purpose and also participate in keeping the environmental sanitation of the area. They also engage in the construction of the health center fences when invited and generating of income for schools through trimming of grasses in the summer for selling and generating income for schools.

Moderator: How about the contribution of the community when it is required to carry mothers and take them to HCs during labor and the likes?

Participant response: yes there is a support of such kind and they support through their labor force, and advice the pregnant mothers to keep their envirnoment and food clean and deliver then to the HC carrying them to the HC during labor.

Participant response: If you ask us about our contribution, well, we advice and educate them starting from the beginning using the education we received.

Moderator: we are at the end of our questions but if you have anything you want to mention and you forget to mention.

Participant response: one thing we forgot to mention is on the provision of bed nets for malaria prevention, which has stopped now but which was used to be given in previous times.

Moderator: Is there any malaria disease threat in the area?

Participant response: no there is no malaria disease in the area unless for some stagnating places which are removed through mobilizing the community to remove the breeding sites and there was also an annual insecticide spay made last year, and there is no malaria any more. The community support us in removing of bushes conducive for malaria breeding.

Participant response: We recommend if we get Bed nets for us the HDAs not to get infected of malaria.

Participant response: the other thing is the HDAs have been registered for getting support in shoes and which is provided to other HDAs in the other ‘Gote’, and we want to ask why is it ours delayed and why the government is not providing it to us as well.

# FGD with mothers GenetAbo_Quarit_12122019

| **I** | **Section I: Identification** | |
| --- | --- | --- |
| 1 | Questionnaire ID | Mothers group (26, 34, 26, 28, 30, and 24 years old) |
| 2 | Area Identification | **Genet Abo** |
| 3 | Name of Woreda | **Quarit** |
| 4 | Name of Kebele |  |
| 5 | Name of moderator | **GT & KA** |
| 6 | Name of note taker |  |
| 7 | Date of discussion |  |
| 8 | Start time: |  |
| 9 | End time: |  |

**Discussion guide**

|  | **For all participants** |
| --- | --- |
| 1 | Interviewer: How early do women go for ANC? How early do women go for PNC? Why do they go at that time? Why earlier or later?  Response: I started after 2 months. Most women started after feeling movement of the fetus-about after 4 months.  R2: We went within 3-4 months depending on the fetal movement.  Interviewer: After delivery, do mothers go for PNC?  Response: They went after 45 days for vaccination. They will not go unless they feel ill or the bay becomes ill.  R2: Ya, they told us to go the 45 days, we went on that day. We went to the HC for vaccination.  Interviewer: Whey they don’t go early?  Response: They started late because they don’t know whether mensus would return or not. Sometimes, mensus would come late after its due.  Interviewer: Why late for PNC?  Response: They went to delay pregnancy. |
| 2 | Interviewer: What kinds of services do they receive in ANC, childbirth and PNC? Are they satisfied?  Response: ANC: Services for the health of the mother and the fetus. Iron, presentation, counseling on feeding.  PNC: care for newborn, vaccination for 45 days, breastfeeding,  Delivery: glucose, yemit merfe, eye ointment…  R2: They helped me well… they diagnosed me with hypertension. They gave me medicine, secure IV line and refer me to Markos. There they gave me medicines, they followed me closely. ….My pressure dropped and they delivered me via CS. They really treated me well. It is also the same here. Everything is available.  As to me we love it. We love the services they are proving us. We are on their help. |
| 3 | Interviewer: Do women think skilled attendance during pregnancy, childbirth, and PNC helps their pregnancy, babies and themselves?  Response: When they feel fatigued, prolonged labor, to complete the follow-up. |
| 4 | Interviewer: If women do not go for ANC, delivery, and PNC what are their reasons?  Explain the obstacles influenced women to utilize ANC, PNC, facility delivery services in your community? Obstacles using different care providers?  Response:  R1 and R2: Easy labor.  R2: It depends on the labor progress. Most women delivered at health facility. We fear the complications. Our neighbor, she was following her pregnancy at the HC. Her labor precipitated and gave birth at home. Unfortunately, she bleeds profoundly. We took her to the HC and the HC referred to Finoteselam. They treated her and she back home alive. It is them they are helping us. We do nothing for such instances.  When we feel healthy, we went 4x. but if we feel ill, we went frequently without dropping out.  R1: Some women fear the fetus would grow and makes the labor long. As such, they stopped following ANC.  Difficulty or unable to travelling on foot, for those lived far.  Interviewer: Why don’t u seek care at facility postpartum?  Response: We felt healthy, we don’t feel sick. They told us to come on 45 days and we went. |
| 5 | What costs do you think involved for pregnant women to attend ANC, facility delivery and PNC services? |
| 6 | 1. Why do women go to the facility for first ANC, but discontinue for subsequent ANC visits? Are all mothers abiding to their appointment dates for ANC consultation? 2. Why do women go to the facility for ANC, yet mostly deliver at home?   Why do women go to the delivery at the facility, yet mostly don’t receive PNC? Are all mothers abiding to their appointment dates for PNC consultation? |
| 7 | Interviewer: In your opinion, what should be improved regarding ANC, facility delivery, and PNC  Response: Generally, they are good. But differs as per our physique. Some are good some are bad. They mentioned instances of disrespect and abuse.  We don’t fear women. But in terms of behavior men are better. We don’t have preferences but behavior.  Most disliked for institutional delivery is the position and vaginal exam. It is very painful.  R2: Nowadays, it becomes public. I don’t like to show for my mother or my ister. Even I don’t like to tell my pregnancy. But now, we were exposed.  We fear the vaginal exam. They also insult or slap if the mother says….  She slped me saying min yargebegibeshal…. |
| 8 | Can you tell us about the traditional practices and beliefs during pregnancy, delivery and postnatal period in your community? |
| 9 | How do you see TBA, health professionals and maternal health services provided to the community? |
|  | **For recently delivered mothers only** |
| 10 | How do you rate the quality of care you received during ANC follow-up, childbirth and PNC? What kinds of services do you receive? Were you satisfied? |
| 11 | Explain factors that would motivate women to utilize ANC service, institutional delivery, and PNC |
| 12 | 1. In your opinion what are reasons for pregnant women not attending ANC services? If women do not go for ANC, what are their reasons? 2. In your opinion what are reasons for pregnant women to deliver outside a health care facility? For home delivered mothers, what do you think are the obstacles when accessing a health care facility? 3. In your opinion what are reasons for pregnant women not attending PNC services? If women do not go for PNC, what are their reasons? |
| 13 | Explain us your experiences relating to the utilization of ANC, birth, and PNC care provided by skilled birth attendants. Prove for;   - their interactions with skilled birth attendants during ANC, delivery, and PNC - their confidence in skilled birth attendants’ abilities, and   respect and compassion of attendants ( respect for the traditional beliefs of the women, etc) |
| 14 | Explain the support you get from the community to and decision making on health services during pregnancy. delivery and postnatal period |
|  | **For community and religious leaders and community volunteers only** |
| 15 | Interviewer: How the community see the maternal health programs and health professionals? Tell me the perception about maternal health care services. Perception about different care providers.  Response: They helped us all rounded household activities. Until we got strong, they helped us. |
| 16 | Interviewer: What efforts your community made to increase maternal health service in your community?  Response: Better behavior. Reduce vaginal exam. |

# FGD with recently delivered mothers-191104_0244_Dembecha

| **I** | **Section I: Identification** | |
| --- | --- | --- |
| 1 | Questionnaire ID | **191104_0244** |
| 2 | Area Identification |  |
| 3 | Name of Woreda | **Dembecha** |
| 4 | Name of Kebele |  |
| 5 | Name of moderator | **CBe** |
| 6 | Name of note taker |  |
| 7 | Date of discussion |  |
| 8 | Start time: | **______:________** |
| 9 | End time: | **49:22** |

| **II** | **Section II: Participant Demographic Intake Sheet** | | | | | |
| --- | --- | --- | --- | --- | --- | --- |
| 1 | **Participant code** | **01** | **02** | **03** | **04** | **05** |
| 2 | Age | 20 | 30 | 25 | 25 | 30 |
| 3 | Religion |  |  |  |  |  |
| 4 | Marital status |  |  |  |  |  |
| 5 | Are you employed? (Yes/No) |  |  |  |  |  |
| 6 | Educational level | 10 | 4 | 0 | 4 | 0 |
| **For recently delivered women** | | | | | | |
| 7 | Gravidity | 0 | 0 | 0 | 0 | 0 |
| 8 | Parity | 1 | 4 | 3 | 3 | 5 |
| 9 | Place of delivery in last pregnancy (facility/home) |  |  |  |  |  |

1. **Practice of ANC, facility delivery, and PNC services**
2. How early do women go for ANC? How early do women go for PNC? Why do they go at that time? Why earlier or later?

Moderator: when did most of you start your ANC visits?

Participant response: As soon as our status with the pregnancy was known.

Moderator: when did you start your first ANC?

Participant response: on my third month of pregnancy.

Moderator: How about the rest of you, for instance you, when did you start your ANC?

Participant response: I started when I was four month pregnant.

Moderator: How about you, when did you start your ANC?

Participant response: I also commence ANC starting from the third month of my pregnancy.

Moderator: why don’t women start their ANC before the three month of pregnancy?

Participant response: this may be because there is the uncertainty and not sure if there is pregnancy before the three month.

Moderator: What else is there for starting on the third month?d

Participant response: we start after the three month because it is also time we started to get sick, loss appetite, or our bodies get fatigued and then we go to the HC to start the ANC. We get tested and if we are tested positive, we will be told of our status and advised to commence with the ANC follow up.

Moderator: Why don’t the women go before the three month after pregnancy?

Participant response: its because they have no any illnesses. Why would we go if we don’t have any pain.

Moderator: What if the pregnancy doesn’t have any pains, do you go?

Participant response: No, we just stay home, we don’t go if we are not sick.

Moderator: for instance, what was the type of pain that made you go to the HC during your pregnancy time?

Participant response: it made me loss appetite, and was accompanied with vomits ***[there was laughter in the room after her explanation that stayed for some time, and then she continued*],** and then after the test, I was told Abiti was conceived.

Moderator: Ok what else, for instance, if you know that you are pregnant and do not feel any pain, do you go to the HC for ANC?

Participant response: we of course go to the HC since we have a vaccination to take every three months as well, that is even if we don’t have any pain, we go every three month based on our schedule.

Moderator: do you all go to the HC, do you use transport and where do you make your ANC follow ups?

Participant response: yes we all to to make ANC, and the use of transport depends, sometimes we use transport and other times we travel on foot. It is Dembecha HC that we go to make the ANC follow up and it will take us 2 hrs to reach it through walking.

1. What kinds of services do they receive in ANC, childbirth and PNC? Are they satisfied?

Moderator: what was the kind of services you received from the HC?

Participant response: well we get services depending on our needs, they give us advice, they correct any abnormalities we might have with the fetus.

Moderator: what else?

Participant response: they tell us to eat a balanced food important for the fetus and advise us also not to engage in any hard works.

Moderator: How about during your delivery, PNC, where did you give birth?

Participant response: I have delivered at the HC at Markos because I was referred.

Moderator: How was the service provided on the delivery, ANC and PNC?

Participant response: the counselling during the ANC and other services was very supportive and on the PNC service I have not encountered any problems. When we come here at the HC, they also advise us properly.

1. Do women think skilled attendance during pregnancy, childbirth, and PNC helps their pregnancy, babies and themselves?

Moderator: How do you see the importance of delivering at the HC with a skilled professional than delivery at home?

Participant response: well when it is at the health we get help to stimulate the labor if we are having prolonged labor and also vaccination for the newborn. If it is at home we will be deprived of this opportunities.

1. **Reasons for use of ANC, facility delivery and PNC**
2. Explain factors that would motivate women to utilize ANC service, institutional delivery, and PNC, **Probe** for reasons for using continuum of care

Moderator: what are the factors that motivate mothers to make the ANC follow up?

Participant response: no pregnant women stays home nowadays without making ANC, all of them are aware of the importance.

Moderator: what will be the factor for most pregnant women to make ANC visits?

Participant response: if she doesn’t make ANC and do not have a registration card ready she will suffer when she goes to the HC for delivery and will not get a very quick service.

Moderator: what else? Is there any other factor that motivates you to go and make ANC?

Participant response: well, there are many hypertensive women and with other conditions and the HEWs advice us to make the ANC. We take the vaccination here at the HP but we take medication from the doctors at the HC.

Moderator: what motivates you to go to the HC to make the ANC?

Participant response: well because they can help us if we are having any unforeseen challenges with the amniotic fluid appearing first during the course of our pregnancy. If we also get weak they support us and for this reason, there is no women who deliver home nowadays.

Moderator: do yo make PNC after you give birth?

Participant response: unless it is for the baby vaccination we don’t make PNC for us. We go to the HC for the baby after the 30 days of birth.

Moderator: Didn’t the HC tell you to come for PNC for you?

Participant response: we haven’t been told by the HC that there is PNC for us, but a vaccination for the baby.

1. **Barriers for attending ANC, facility delivery and PNC use**
2. If women do not go for ANC, delivery, and PNC what are their reasons?Explain the obstacles influenced women to utilize ANC, PNC, facility delivery services in your community? Obstacles using different care providers?, **Probe** for financial barriers and opportunity costs, geographic barriers, socio-cultural barriers and quality of care barriers.

Moderator: why do you think other women don’t go to the HC for ANC?

Participant response: I think there are no women who doesn’t make ANC now a days, I for instance gave I birth to twins two years back at home, and the next delivery I couldn’t make it at home and delivered at the HC because of, I think, the shingles disease I developed at the time, so sometimes it is not our call and the labor may come sooner than the due date. If the pregnant women are sick after delivery, the postpartum women are admitted at the HC for sometime and if they are well, they will be send back to their homes with an ambulance, for instance, I refused to be admitted and requested to be taken back home and was taken with an ambulance.

Moderator: ***[leading question for the above respondent],*** for instance you have delivered the baby you are holding now in your home, what was your reason?

Participant response: well it is because I was not sick. I was feeling pain sometime before and seek medication and I was appointed to come for delivery, however, as I was planning to go to the HC, the labor came unexpectedly and mean time before the appointment day and it forced me to deliver home.

Moderator: Didn’t you want to call an ambulance and go to the HC, and who delivered you home?

Participant response: No I didn't want to call an ambulance, and I delivered it on my own that is with my family who had experience in delivering.

Moderator: can it be said for you not to deliver at the HC was because you have a nature in having quick deliveries once your labor starts?

Participant response: yes, unless for the associated pains, labor does not stay long on me, and I was able to deliver in less than 1 hour after my labor started.

Moderator: what else do the women in this area mention as a factor for not delivering at the HC?

Participant response: there are women who says the HC is not up to the level to deliver them and who as a result visit other HFs. There are many women who does not want to deliver in this HC and prefers a referral to Markos for making an operation and for a normal delivery rather.

Participant response: the service provided by Dembecha and at Markos is not the same. For instance, when I come once to this HC for making ANC, the professional told me the fetus was fine, and including its positioning and that they are going to deliver me at the HC, and when I went the second time after I encountered unexpected labor, he ask me if my home was near to the HC, and to go and have some coffee if I have any relatives around and that they are going to admit and deliver me later. And another professional with better competence came and see me, and commented the previous professional if he is trying to get me killed delivering here and also on the way to Markos if am not referred soon, and then he intervened to make a referral to two pregnant women to Markos, send all of us three; two pregnant women and one patient with an accident within a single ambulance to Markos. Generally, I don’t think there is a competent health professional at Dembecha HC at all. I would have died just like he commented if I was not referred to Markos timely. The later health professional who attended me second regretted that I was not referred timely.

Participant response: In my case, they didn’t tell me that I was having twins here at Dembecha HC, but I was aware and knew my status because of my previous experience. The health professionals attend better the people coming from the urban areas and not us who are coming from the rural area which they have poor consideration. After I arrived at Markos after the referral, they tried to inform me that I was having twins with manners and mocked Dembecha HC’s ANC follow up feedback to me that they said my pregnancy was fine this whole time and that they didn’t notice the presence of twins. Dembecha HC plays with the pregnant mothers and attends with students for apprenticeship and then refers pregnant women when they get critical.

Participant response: they give more attention when they see educated people and not us the rural dwellers, and some pregnant women remains home during their labor because of this. Majority women question what would the health professional do for them other than sitting out their time, enjoying rather than attending them during their labor and most remain home during their labor for this reasons.

Moderator: What other problems are there other than the problems you told me, may be lack of money or the distance?

Participant response: It is not that, the professionals don’t attend us, the pregnant women say what would the professionals do to them if they go during labor since they don’t attend them and hence, would prefer to remain home for it has no difference coming at the HC and would rather prefer remaining home since they give attention to the educated ones. Just recently, Melaku’s wife in my neighbour went to the health center and has been suffering the whole day and no one was there to attend her and when the people accompanying her requested the midwifes to come and see her, they were saying she is still in her early labor and suggested to wait for sometime, but the other experienced health professional arrived and attended her. But in my case, I delivered at the same time I arrived at the HC’s gate.

Moderator: Would the distance be a factor for not using the HC during your labor?

Participant response: no there is an ambulance assigned to us and we call Tigist when we need the ambulance and Tigist calls the ambulance for us and Ambulance is not that an issue. It is is in deed small in number, but there is also a Bajaj transport service if we need to use.

Participant response: there is a shortage in the ambulance service, for instance, a women in the neighbour has been calling them the whole night once during her labor but she was only sent early in morning using this ambulance, and generally, the ambulance service does not function in the night since they get bored and not available. Ambulance is not easily available.

1. **Reasons for discontinuation**
2. Why do women go to the facility for first ANC, but discontinue for subsequent ANC visits? Are all mothers abiding to their appointment dates for ANC consultation?

Participant response: no they don’t discontinue their ANC once they start it. After we have our registration we go back to the HC and don’t dis continue, and I remember once it was holiday on the day of my appointment and considered going to the HC one day before but the midwife asked why I did come that day when my appointment was after one day and send me back, I don’t know how one day makes a difference in the ANC but thanks to that one professional who called me back and examined me.

1. Why do women go to the facility for ANC, yet mostly deliver at home?

Participant response: women might go to the HCs for delivery if it is mandatory, but most would say why would we go to deliver at the HC, for there is little the HC would do to help them. Most make their ANC follow ups but during delivery they try it with TBAs since they are not satisfied with the delivery service at the HC.

1. Why do women go to the delivery at the facility, yet mostly don’t receive PNC? Are all mothers abiding to their appointment dates for PNC consultation?, **Probe** for financial barriers and opportunity costs, geographic barriers, socio-cultural barriers and quality of care barriers
2. What costs do you think involved for pregnant women to attend ANC, facility delivery and PNC services?

Moderator: Is there any expenses enquired of you during your delivery?

Participant response: No, there was not any expenses during my delivery, since there are also no medication prescribed during pregnancy.

Moderator; for instance, after your delivery does that ambulance that took to the HC return you back to your homes after delivery, is there any costs related with this?

Participant response: No, they don’t return us back home, and we spend 100 ETB for Bajaj transport for returning home. There is also a problem even with taking us to the HC during our labour.

1. In your opinion, what should be improved regarding **ANC, facility delivery, and PNC?**

Participant response: regarding the ANC, the professionals don’t conceal what they know to tell us, but regarding the delivery service, it is better if they can serve better and if they don’t have the capacity refer us timely and if they have the capacity, they have to serve us obediently rather than being showy in the compound. It is only the farmers who stick here for the services but those educated seek for different options in the different private institutions.

Moderator: what else do you think should be improved about their services?

Participant response: they don’t endeavor to provide us with our results on timely manner, instead they relax and enjoy their times while we are sited, waiting and eager to return to our homes. There are some of us coming from distant places and they might appoint you in the afternoon, but we might have our kids to feed and sometimes forced to leave without knowing our test results.

Participant response: there was a time which once I forgot to bring my registration card when I visited the HC and which the health professional send me back commenting why I did I come in the first place if I don’t have my car with me. When I ask him that my village was far to go and bring back the cards and to examine me instead since my pregnancy is already confirmed, he still commented I should not have come, which then I went out and never returned to the HC again. Unless it is for the lack of willingness by the professionals, there is an option to re-discover the replacement registration card from the HC.

Participant response: there is generally shortage of adequate ambulance and also interrupting our services by the health professionals to provide the service if a wife of a government employee comes for ANC, and not serving us well based on the order of our arrival. They also wait until we get weak and critical during our labor and refer us after.

Moderator: what about shortage of drug?

Participant response: they give us mostly the ampicillin whenever we are sick but they have drugs that they give us and it is not a challenge.

1. **Traditional practices during pregnancy, childbirth and postnatal period**
2. Can you tell us about the traditional practices and beliefs during **pregnancy, delivery and postnatal period** in your community?

Moderator: is there anything that is practiced in this locality for a women after her delivery or while she was pregnant, any cultural practices?

Participant response: there is no practice related with FGM or cultural practice of cutting of tonsils for children nowadays.

Moderator: How about gun-firing ceremony when a women delivers a baby?

Participant response: no there is not, it was in the earlier times; there was practice of making of gunfire for commemorating the birth, and also postpartum taken out of the house, to the backyard by cutting out section of the house, but those were in earlier times and it is no more a practice nowadays, and if she is sick she is taken to the HC, and there is no such cultural practices.

1. Do you think these traditional beliefs, religious practices, and cultural norms affect mothers to use care during pregnancy, delivery, and postpartum period in your community? Explain how and why?
2. How do you see community volunteers/TBAs, health professionals and maternal health services provided to the community?

Moderator: how do you see the support provided by the health extension workers and TBAs to you?

Participant response: well, they vaccinate us, provide us with education and advise us to go seek medication and keep our personal hygiene.

**For Recently Delivered Mothers Only.**

1. Explain factors that motivate you to utilize ANC service, institutional delivery, and PNC, **Probe** for reasons for using continuum of care
2. In your opinion what are reasons for not attending ANC services?
3. For home delivered mothers, what do you think are the obstacles when accessing a health care facility? **Probe** for discontinuation after receiving ANC, If women do not go for PNC, what are their reasons? **Probe** for discontinuation after receiving ANC and/or facility delivery.
4. Explain us your experiences relating to the utilization of ANC, birth, and PNC care provided by skilled birth attendants. Prove for;

- their interactions with skilled birth attendants during ANC, delivery, and PNC
- their confidence in skilled birth attendants’ abilities, and respect and compassion of attendants ( respect for the traditional beliefs of the women, etc)

1. Explain the support you get from the community to and decision making on health services during **pregnancy. delivery and postnatal period.**

Participant response: it is only food we get from our community or family when we are still in the HC after delivery. It is mainly our families that support us during our pregnancy and through an advice as well.

Moderator: is there anything that you finally want to add?

Participant response: there is nothing we didn’t mention but to correct the treatment of the professionals towards us and to avail us the ambulance service timely by the HC. It would be great if they can improve on these.
